# Vitamin D supplementation in infertile men: a systematic review and meta-analysis of effects on semen quality and endocrine function

**DOI:** 10.7717/peerj.21002

**Published:** 2026-04-13

**Authors:** Guoqing Zhang, Giribabu Nelli, See Ziau Hoe, Mukhri Hamdan

**Affiliations:** 1 Department of Obstetrics & Gynaecology, Faculty of Medicine, Universiti Malaya, Kuala Lumpur, Malaysia; 2 Department of Physiology, Faculty of Medicine, Universiti Malaya, Kuala Lumpur, Malaysia

**Keywords:** Male infertility, Vitamin D supplementation, Semen parameter, Reproductive hormone, Pregnancy outcome

## Abstract

**Objective:**

To evaluate the effects of oral vitamin D supplementation on semen parameters, reproductive hormones and fertility outcomes in infertile men.

**Methods:**

We searched PubMed, Cochrane Library, Web of Science, Scopus and Embase for studies published within the last 10 years. Randomized controlled trials (RCTs) and observational studies evaluating oral vitamin D supplementation in infertile men and reporting semen parameters, reproductive hormones and/or fertility outcomes were included. Risk of bias was assessed using RoB 2.0 (RCTs) and ROBINS-I (observational studies).

**Results:**

Eleven studies (eight RCTs and three observational studies; *n* = 1,168) were included. Vitamin D supplementation was associated with small but statistically significant improvements in semen volume, sperm concentration, progressive motility and serum testosterone. No significant effects were observed on clinical pregnancy or live birth. Reporting of adverse events was limited; no vitamin D-related serious adverse events were reported.

**Conclusions:**

Vitamin D supplementation may improve selected semen parameters and testosterone levels in infertile men, particularly progressive sperm motility. However, current evidence does not demonstrate improved clinical fertility outcomes, and higher doses (>4,000 IU/day) do not appear to confer additional benefits. Larger, well-designed trials with standardized fertility endpoints are needed to confirm clinical efficacy.

## Introduction

Infertility is defined as the inability to achieve pregnancy after 12 months of regular unprotected intercourse. In recent decades, the global prevalence of infertility has risen significantly, posing a major public health concern (Feng et al., 2025; Liang et al., 2025). Although the paternal effect is less pronounced compared to the maternal influence, male factors contribute to infertility in approximately 30–50% of affected couples (Eisenberg et al., 2023).

Bibliometric analyses have revealed an exponential growth in research on male infertility over the last two decades (Baskaran et al., 2021; Zhang et al., 2016), reflecting both a growing recognition of the importance of reproductive health and rapid advancements in the field of male infertility research. However, the clinical management of male infertility remains a significant challenge, particularly due to the prevalence of sperm abnormalities such as oligozoospermia and asthenozoospermia which are commonly observed in clinical practice (Jiao, Yang & Chen, 2021).

In addition to sperm-related issues, hormonal imbalances, particularly steroid hormone deficiencies, play a critical role in impairing male reproductive function (Jiao, Yang & Chen, 2021). Given the intricate interplay between nutrition, hormonal regulation, and spermatogenesis, dietary factors and nutrient balance have emerged as important modifiable elements in the management of male infertility (Pecora et al., 2023; Suliga & Gluszek, 2020; Torkel et al., 2024). Key nutrients, including antioxidants, essential fatty acids, zinc, selenium, and vitamins (*e.g.*, vitamins C, E, and D), have been implicated in sperm function, hormonal regulation, and overall fertility potential (Salas-Huetos, Bullo & Salas-Salvado, 2017; Salas-Huetos et al., 2019). Among these, vitamin D has attracted considerable attention for its potential role in regulating spermatogenesis (Sood et al., 1992; Sood et al., 1995; Sun et al., 2015) and steroid hormone production (Lambeth & Stevens, 1984; O’Shaughnessy & Murphy, 1991).

Vitamin D is a fat-soluble vitamin involved in calcium homeostasis, immune regulation, and other physiological processes (Giustina et al., 2024). The vitamin D receptor (VDR) and key vitamin D metabolizing enzymes are expressed in testis tissue and mature sperm (Boisen et al., 2021; Hu et al., 2023; Wang et al., 2022; Xue et al., 2022). In VDR knockout mice, sperm counts and motility are reduced and fertility is impaired (Kinuta et al., 2000), supporting an essential role for vitamin D signaling in male reproductive function.

Evidence from clinical studies further supports this mechanistic link. Serum vitamin D levels have been found relatively lower in infertile men compared to fertile counterparts (Rezayat et al., 2022). Furthermore, vitamin D deficiency in adult males has been associated with reduced semen quality (Özkaya & Demirel, 2018). This bidirectional relationship between vitamin D status and reproductive health has prompted growing interest in the potential therapeutic value of vitamin D supplementation for male infertility—particularly regarding its effects on semen parameters, hormonal profiles, and clinical fertility outcomes.

However, clinical evidence regarding the benefits of vitamin D supplementation in infertile men remains inconsistent. For instance, some studies demonstrated that vitamin D supplementation improves sperm concentration and progressive sperm motility (Alzoubi et al., 2017; Bartl et al., 2021), while some studies failed to demonstrate significant effects, particularly on progressive sperm motility (Blomberg Jensen et al., 2018; Gheflati et al., 2021). To our knowledge, no previous meta-analysis has comprehensively assessed semen parameters, sex steroid hormones, and fertility outcome.

To bridge this gap, we conducted a systematic review and meta-analysis of studies published in the past decade. Our objectives were to quantify the effects of vitamin D on semen parameters and reproductive hormones, explore heterogeneity through dose- and duration-stratified analyses, and provide evidence-based recommendations for clinical practice.

## Materials & Methods

This systematic review and meta-analysis was prospectively registered in the International Prospective Register of Systematic Reviews (PROSPERO; Registration No. CRD420251017427). The review was conducted in accordance with the PRISMA 2020 and followed methodological recommendations from the Cochrane Handbook for Systematic Reviews of Interventions (Higgins et al., 2019), including both randomized controlled trials (RCTs) and observational studies.

### Research objective

The primary objective of this study was to evaluate whether vitamin D supplementation improves reproductive health outcomes in infertile adult men. Specifically, we assessed the effects of vitamin D on semen parameters, reproductive hormone profiles and clinical fertility outcomes.

### Eligibility criteria

Following the PICOS (population, intervention, control, outcomes, study design) question, we included.

Population (P)

The study population consisted of adult male patients diagnosed with infertility and seeking treatment at a reproductive center.

Intervention (I)

The intervention of interest was oral vitamin D supplementation, either as monotherapy or in combination with medications.

Comparison (C)

For randomized controlled trials, the comparison group consisted of infertile male patients receiving an oral placebo. In observational studies, the comparison was established through self-controlled designs, where participants served as their own controls through pre- and post-intervention assessments.

Outcomes (O)

The primary outcomes were semen parameters (including semen volume, sperm concentration, total sperm count, progressive sperm motility, and morphology) and clinical pregnancy outcomes. Secondary outcomes included changes in serum steroid hormone profiles, specifically measuring testosterone (T), follicle-stimulating hormone (FSH), and luteinizing hormone (LH) levels through standardized laboratory assays.

We excluded following studies: (1) included populations with major systemic diseases (*e.g.*, diabetes, chronic liver/kidney disease, malignancy, endocrine disorders), organ transplantation, women/child/adolescent/non-human/*in vitro* studies; (2) used interventions known to independently affect spermatogenesis or hormonal profiles (*e.g.*, zinc supplementation); (3) lacked an appropriate comparators; or (4) did not report relevant semen, hormonal or fertility outcomes, or provided insufficient data for meta-analysis. Only full-text articles published in English were included.

### Search strategy

To ensure a comprehensive search strategy, we initially searched the Medical Subject Headings (MeSH) database using the keywords “male infertility” and “vitamin D” to identify relevant MeSH terms. Subsequently, we developed and executed a systematic search strategy across five major electronic databases: PubMed, Cochrane Library, Web of Science, Scopus, and Embase.

The search strategy incorporated a combination of MeSH terms and free-text keywords, with appropriate Boolean operators and truncations adapted to each database’s specific requirements. The search strategy used in different databases is included in Table 1.

To ensure the inclusion of the most recent and relevant studies, the search was restricted to ten years (March 3, 2015–October 3, 2025). Although no formal kappa statistic was calculated, consensus was achieved through discussion or involvement of a third reviewer. The retrieved literature was organized and managed using EndNote, enabling efficient sorting and citation management.

### Screening process

Following the initial literature retrieval, all records were uploaded to the Rayyan platform to systematically identify and remove duplicate studies, thereby streamlining the screening process (Ouzzani et al., 2016). The literature screening was first conducted by two independent reviewers (Guoqing Zhang and Nelli Giribabu) based on the abstract and title. Discrepancies were resolved by consensus or consultation with a third reviewer (Hoe See Ziau). The full-text evaluation was carried out and listed the reasons for exclusion. All exclusion decisions were listed with specific reasons and recorded following Preferred Reporting Items for Systematic Reviews and Meta-Analyses (PRISMA) guidelines.

### Data extraction

Data extraction was conducted meticulously by two independent reviewers in a blinded manner (Guoqing Zhang and Nelli Giribabu). Key study characteristics, including study country, publication year, sample size, average ages, research design, and intervention period, were systematically collected. Additionally, data on sperm parameters and steroid hormone levels were extracted in a structured manner. As these variables are continuous, the mean and standard deviation (SD) were obtained for analysis. In cases of missing data, appropriate statistical techniques were applied by two independent reviewers (Guoqing Zhang and Nelli Giribabu) to ensure data integrity and maintain the robustness of the dataset. Discrepancies in data extraction were resolved through discussion, and if disagreement persisted, a third reviewer (Hoe See Ziau) was consulted to reach a consensus.

**Table 1:** Search strategies used in different databases.

#	Search term
PubMed	(male infertility OR aspermia OR asthenozoospermia OR azoospermia OR oligospermia OR “sertoli cell-only syndrome” OR teratozoospermia) AND (vitamin D OR cholecalciferol OR hydroxycholecalciferols OR ergocalciferol OR “25-Hydroxyvitamin D2” OR dihydrotachysterol)
Web of science	((ALL= (male infertility) OR ALL=(Aspermia) OR ALL=(Asthenozoospermia) OR ALL=(azoospermia) OR ALL=(Oligospermia) OR ALL= (sertoli cell-only syndrome) OR ALL=(Teratozoospermia)) AND (ALL= (vitamin D) OR ALL=(cholecalciferol) OR ALL= (hydroxycholecalciferols) OR ALL=(Ergocalciferol) OR ALL= (25-Hydroxyvitamin D2) OR ALL=(Dihydrotachysterol)))
Cochrane Library	(male infertility OR aspermia OR asthenozoospermia OR azoospermia OR oligospermia OR “sertoli cell-only syndrome” OR teratozoospermia) AND (vitamin D OR cholecalciferol OR hydroxycholecalciferols OR ergocalciferol OR “25-Hydroxyvitamin D2” OR dihydrotachysterol)
Scopus	(“male infertility” OR aspermia OR asthenozoospermia OR azoospermia OR oligospermia OR “sertoli cell-only syndrome” OR teratozoospermia) AND (“vitamin D” OR cholecalciferol OR hydroxycholecalciferols OR ergocalciferol OR “25-Hydroxyvitamin D2” OR dihydrotachysterol))
Embase	(“male infertility” OR aspermia OR asthenozoospermia OR azoospermia OR oligospermia OR ‘sertoli cell-only syndrome’ OR teratozoospermia) AND (‘vitamin D’ OR cholecalciferol OR hydroxycholecalciferols OR ergocalciferol OR ‘25-Hydroxyvitamin D2’ OR dihydrotachysterol)
Publication Years	Ten-year period (March 3, 2015 –October 3, 2025)

### Risk of bias assessment

The methodological quality and risk of bias of the included studies were rigorously assessed using the Risk of Bias (ROB) tools recommended by the Cochrane Handbook (Higgins et al., 2019). Two independent reviewers (Guoqing Zhang and Nelli Giribabu) conducted the evaluation process, with any discrepancies resolved through consultation with a third reviewer (Hoe See Ziau).

For RCTs, the risk of bias was assessed using the ROB 2.0 tool recommended by the Cochrane Handbook. The assessment encompassed five critical domains: (1) random sequence generation, (2) allocation concealment, (3) blinding of participants and personnel, (4) incomplete outcome data, (5) selective reporting and other potential sources of bias. Each domain was judged as ‘low risk’, ‘high risk’, or ‘unclear risk’ according to the Cochrane Handbook guidelines. The final evaluation results are presented through the risk of bias summary table and the risk of bias figure.

For observational studies, the risk of bias was evaluated using the Risk Of Bias In Non-randomized Studies—of Interventions tool (ROBINS-I), which assessed seven domains: (1) confounding, (2) selection bias, (3) classification of interventions, (4) deviations from intended interventions, (5) missing data, (6) measurement of outcomes, and (7) selection of the reported result. Each domain was judged as ‘low risk’, ‘moderate risk’, ‘serious risk’, or ‘critical risk’.

### Statistical analysis

Data were synthesized using RevMan 5.4. Continuous outcomes (semen parameters and hormone levels) were summarized as mean difference (MD) with 95% confidence interval (CI). Dichotomous outcomes (clinical pregnancy and live birth) were summarized as risk ratios (RRs) with 95% CIs. Two-sided *P* values < 0.05 were considered statistically significance.

Data analysis methods were guided by the meta-analysis guidelines in the Cochrane Handbook (Higgins et al., 2019). We employed both random-effects and fixed-effects models for data synthesis, selecting the appropriate model based on heterogeneity (I^2^). A random-effects model was used when heterogeneity was high (I^2^ > 50%), while a fixed-effects model was applied when heterogeneity was low (I^2^ ≤ 50%).

When heterogeneity was high (I^2^ > 75%), we explored potential sources using prespecified subgroup analyses by treatment duration (≤3 months *vs* 5–6 months) and daily dose (4,000 IU/day *vs* > 4,000 IU/day), and conducted leave-one-out sensitivity analyses to assess the influence of individual studies. To further explore the heterogeneity that could not be explained by subgroup analysis and sensitivity analysis, we performed both frequentist and Bayesian meta-regression analyses. Weighted linear regression was used for initial exploration, while Bayesian modeling allowed for probabilistic interpretation of covariate effects. Predictor variables (baseline of 25(OH)D and body mass index (BMI)) were standardized for comparability. Detailed modeling procedures, priors, and diagnostics are described in the Supplementary Methods. All analyses were conducted in R 4.3.1 with brms and metafor packages.

## Results

### Study selection

**Figure 1:**
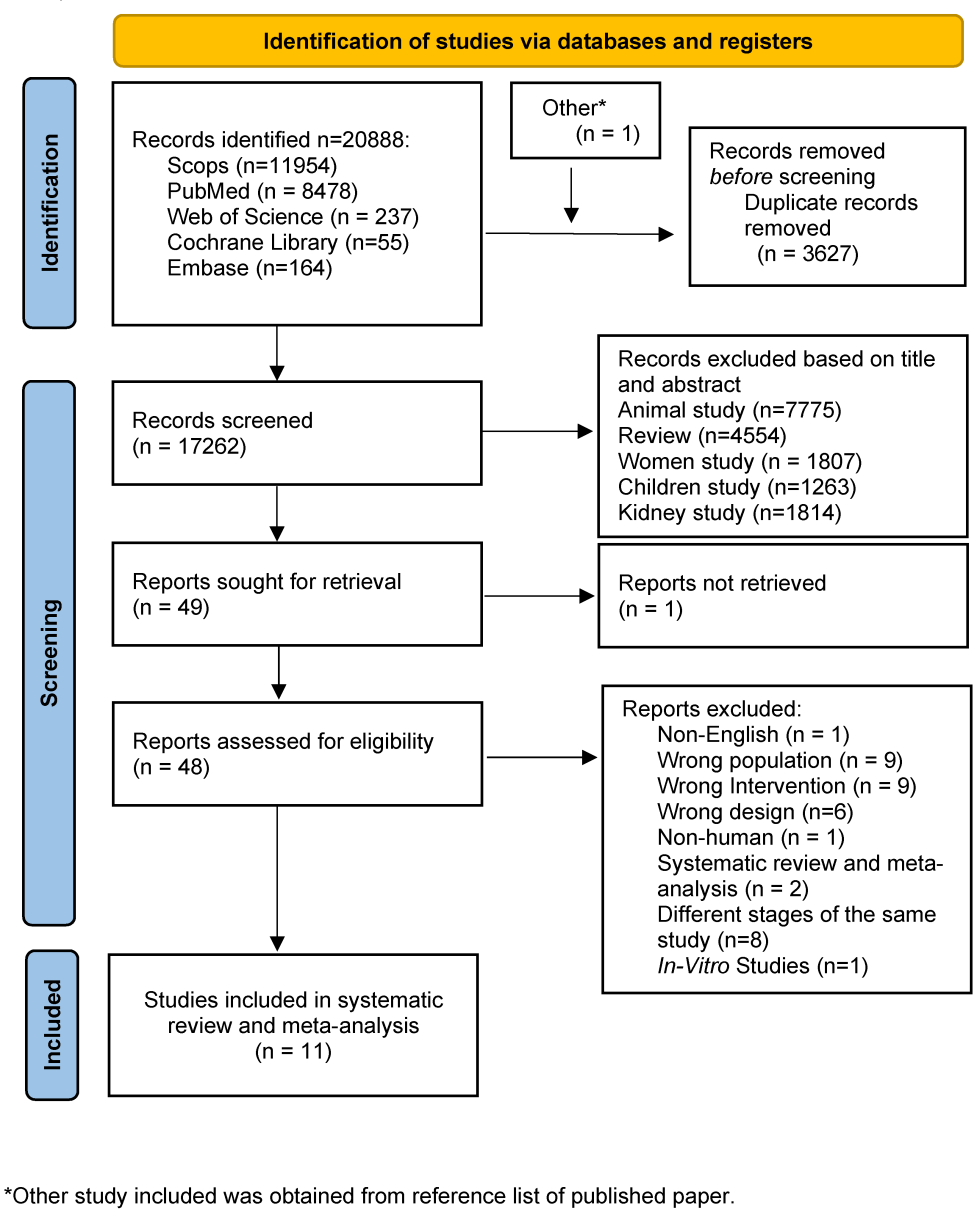
Flow diagram of the study selection process.

The study selection process is summarized in Fig. 1. We indentified 20,888 records across databases. After removing 3,627 duplicate, 17,262 records were screened by title/abstract and 48 full-text articles were assessed. Eleven studies met the inclusion criteria and were included in the qualitative synthesis and meta-analysis.

An initial title screen was conducted to effectively exclude clearly irrelevant publications. We excluded the following types of studies: review articles (*n* = 4,554), animal experimental studies (*n* = 7,775), research specific to women’s health (*n* = 1,807), child and adolescent studies (*n* = 1,263), and research related to kidney diseases (*n* = 1,814).

Of 49 reports sought for full-text retrieval, one could not be obtained. Among the 48 full texts assessed, 37 were excluded for reasons including non-English language (*n* = 1), wrong population (*n* = 9), wrong intervention (*n* = 9), inappropriate study design (*n* = 6), non-human studies (*n* = 1), systematic review/meta-analysis (*n* = 2), duplicate or overlapping reports (*n* = 8), and in-vitro studies (*n* = 1).

Eleven studies were included in qualitative synthesis and meta-analysis.

### Study and participating characteristics

Key characteristics and detailed descriptions of all research included in this study are summarized in Table 2. A total of 11 studies were included in the final analysis, comprising three observational studies and eight RCTs (Alzoubi et al., 2017; Amini et al., 2020; Banks et al., 2021; Bartl et al., 2021; Blomberg Jensen et al. 2018; Gheflati et al., 2021; Holt et al., 2024; Maghsoumi-Norouzabad et al., 2021; Maghsoumi-Norouzabad et al., 2022; Padmapriya et al., 2022; Wadhwa et al., 2020). Among the RCTs, two studies conducted by the same author focused on different outcome measures following the intervention. Regarding the geographical distribution, four studies were from Iran, two from Denmark, two from India, one from Slovakia, one from Jordan, and one from the United States.

**Table 2:** Characteristics of included studies.

**Study**	**Sample size**	**Study design**	**Population**	**Baseline 25(OH)D (ng/ml)**	**BMI (Kg/m** ^**2**^ **)**	**Ages**	**Interventions**	**Control group**	**Outcomes**
Alzoubi et al., 2017, Jordan	45	Observation study	Idiopathic infertile men with low vitamin D	12.56 ± 0.78	–	31.9 ± 0.8	Vitamin D3 5,000 IU, once daily for 2 months	Using self- comparison	3
Gheflati et al., 2021, Iran	44	Double-blind RCT	Men diagnosed with asthenozoospermia	6.81 ± 0.68	25.4 ± 1.4	32.6 ± 1.3	Vitamin D 50,000 IU once a week for 12 weeks	Placebo	1, 2, 3, 4, 9, 10
Padmapriya et al., 2022, India	80	Double-blind RCT	Men diagnosed with OAT (oligoasthenoteratozoospermia)	–	27.1 ± 4.9	33.9 ± 7.8	Vitamin D3 (cholecalciferol) twice a day (4,000 IU) for 72 days	Placebo	1, 2, 7, 8
Bartl et al., 2021, Slovakia	34	Observation study	OAT men with sperm concentration more than 1 million/ml.	24.40 ± 9.02	29.1 ± 3.18	36.6	Cholecalciferol 2,500 IU/day for 6 months.	Using self- comparison	2, 3, 4, 7, 8, 9
Amini et al., 2020, Iran	62	Triple-blind RCT	Infertile men who undergone treatment in fertility clinic	16.67 ± 6.91	25.6 ± 1.9	34.6 ± 4.7	Vitamin D3 50,000 IU once a week for 8 weeks, the remaining 4 weeks (50,000 IU supplement monthly)	Placebo	1, 2, 3, 4, 7, 8, 9, 10,
Maghsoumi-Norouzabad et al., 2021, Iran (1)	86	Triple-blind RCT	Infertile men (the motility of sperm < 40% and rapid progressive sperm motility < 32%) and vitamin D3 levels < 30 ng/ml	18.74 ± 6.62	28.27 ± 2.7	34.7 ± 5.2	Cholecalciferol 4,000 IU once a day for 3 months	Placebo	1, 3, 4,
Maghsoumi-Norouzabad et al., 2021, Iran (2)	86	Triple-blind RCT	Infertile men (the motility of sperm < 40% and rapid progressive sperm motility <32%) and vitamin D3 levels <30 ng/ml	18.74 ± 6.62	28.2 ± 2.7	34.7 ± 5.2	Cholecalciferol 4,000 IU once a day for 3 months	Placebo	1, 3, 4, 5, 7, 8, 9, 10,
Blomberg Jensen et al., 2018, Denmark	296	Triple-blind RCT	Infertile men	14.0 ± 4.4	26.5 ± 4.2	34.8 ± 6.6	Cholecalciferol 300,000 IU initially, then 1,400 IU cholecalciferol and 500 mg of calcium daily for 150 days.	Placebo	1, 2, 3, 4, 6, 7,
Banks et al., 2021, United state	154	Double-blind RCT	Males with sperm concentration between 5 M/mL and 15 M/mL, motility ≤ 40%, or normal morphology ≤ 4% were eligible	–	27.8 ± 5.0	33.7 ± 4.9	Vitamin D3 2,000 IU daily for 6 months	Placebo	5, 6,
Holt et al., 2024, Denmark	307	Triple-blind RCT	Infertile men	18.2 ± 8.0	26.4 ± 4.4	35.0 ± 6.5	Cholecalciferol 300,000 IU initially, then 1,400 IU cholecalciferol and 500 mg of calcium daily for 150 days.	Placebo	7, 8, 10,
Wadhwa et al., 2020, India	60	Observation study	Infertile men with either oligozoospermia or asthenozoospermia with vitamin D levels <30 ng/ml	19.08 ± 4.98	23.0 ± 2.4	30.6 ± 4.0	Cholecalciferol 60,000 IU weekly and 500 mg daily calcium supplementation for 6 months	Using self- comparison	2, 7, 8, 10,

**Notes.**

a Outcomes: 1, Semen Volume; 2, Sperm Concentration; 3, Progressive Sperm Motility; 4, Normal Sperm Morphology; 5, Clinical Pregnancy Rate; 6, Live Birth Rate; 7, FSH; 8, LH; 9, Sex Hormone-Binding Globulin (SHBG); 10, T.

The duration of vitamin D supplementation included in this study was categorized as ≤3 months (Alzoubi et al., 2017; Amini et al., 2020; Gheflati et al., 2021; Maghsoumi-Norouzabad et al., 2021; Maghsoumi-Norouzabad et al., 2022; Padmapriya et al., 2022; Wadhwa et al., 2020) or 5–6 months (Banks et al., 2021; Bartl et al., 2021; Blomberg Jensen et al., 2018; Holt et al., 2024; Wadhwa et al., 2020) time points, considering the complete cycle of human sperm development (from spermatogonia to mature spermatozoa) typically requires approximately 64 to 74 days (Johnson, Petty & Neaves, 1983). One study reported effect sizes at both the ≤3 month and 5–6 months (Wadhwa et al., 2020).

According to the Institute of Medicine, the tolerable upper intake level (UL) for vitamin D in healthy adults is 4,000 IU/day, beyond which the risk of adverse effects may increase (Ross et al., 2011). We stratified studies into≤4,000 IU/day (Banks et al., 2021; Bartl et al., 2021; Blomberg Jensen et al., 2018; Holt et al., 2024; Maghsoumi-Norouzabad et al., 2021; Maghsoumi-Norouzabad et al., 2022) and >4,000 IU/day (Alzoubi et al., 2017; Amini et al., 2020; Gheflati et al., 2021; Padmapriya et al., 2022; Wadhwa et al., 2020). Studies vary widely in both dose and duration, with some trials using weekly high-dose regimens and others employing moderate daily doses over longer periods (Fig. S1).

Among the included studies, nine defined the inclusion criteria for infertile men as a sperm concentration of less than 15 million/ml, or total sperm motility of less than 40%, or progressive motility of less than 32%, and/or normal sperm morphology of less than 4%. One study uniquely adopted distinct diagnostic criteria, defining abnormal semen parameters as sperm concentration below 20 million/ml, and/or progressive motility less than 50%, and/or normal sperm morphology below 12%, with their study cohort comprising a substantial proportion (18%) of hypogonadism participants (Holt et al., 2024). The mean age of participants across all included studies ranged from 30 to 35 years. The mean BMI of participants across all included studies ranged from 20 to 30. No toxicity or side effects were reported in all studies.

### Definition of outcome

Vitamin D status is classified by the Endocrine Society clinical practice guidelines as deficiency (<20 ng/mL or <50 nmol/L), insufficiency (20–29 ng/mL or 50–74 nmol/L) based on serum 25(OH)D levels (Holick et al., 2011).

Clinical pregnancy and live birth outcomes were extracted as defined in the original studies. Specifically, clinical pregnancy was defined variably across trials, including a positive pregnancy hormone test during the study period or participant-reported pregnancy. Live birth was defined as the delivery of a live infant after 20 weeks’ gestation or according to the authors’ reported definitions when explicit criteria were not provided.

### Risk of bias

The overall risk of bias for RCTs (*n* = 8) was predominantly low to moderate (Fig. S2A). Among observational studies (*n* = 3), two had a moderate risk and one showed a high risk, primarily due to insufficient control of confounding factors (Fig. S2B).

Among all included studies, only one (Alzoubi et al., 2017) was assessed as having a high risk of bias. This study did not report participants’ BMI and had notable missing data. It contributed effect estimates for progressive sperm motility to our meta-analysis. The absence of BMI data limits our ability to explore potential effect modification by adiposity, and the methodological limitations may have contributed to the observed heterogeneity in the pooled results. As such, we performed sensitivity analyses to evaluate the influence of this study and interpreted its contribution with caution.

### Sensitive analysis

This study evaluated a total of 12 clinical outcome measures, among which eight exhibited significant heterogeneity (I^2^ > 75%). Initial subgroup analyses based on intervention duration and dosage did not effectively reduce the heterogeneity. Subsequently, higher heterogeneity was preferentially excluded (Alzoubi et al., 2017), and then a stepwise exclusion analysis on measures was conducted, including semen volume, sperm concentration, progressive sperm motility, normal sperm morphology, SHBG, and T. This successfully reduced heterogeneity in six of these outcomes. Notably, the clinical pregnancy rate was not subjected to exclusion analysis as it was reported in only two studies.

The statistical analysis of semen volume, conducted through a stepwise exclusion method for sensitivity analysis, revealed that after excluding the study of Gheflati et al. (2021), the pooled effect size remained largely unchanged, while the heterogeneity I^2^ decreased from 89% to 71%. Consequently, this study was excluded from the final pooled analysis for this outcome. For the analysis of sperm concentration, after excluding the study of Wadhwa et al. (2020), the heterogeneity I^2^ decreased from 96% to 5%.

Despite subgroup analyses by dosage and treatment duration, significant heterogeneity persisted (I^2^ > 50%) across the seven progressive sperm motility studies (Figs. S3A and SB). As sensitivity analysis proved ineffective, we conducted meta-regression to investigate potential moderators, including baseline characteristics.

In the analyses of normal sperm morphology, serum SHBG, and T levels, the exclusion of the study by Gheflati et al. (2021) significantly impacted the results: for normal sperm morphology (pooled effect size: from 2.50 to 9.68; I^2^: from 98% to 67%), SHBG (pooled effect size: from 1.49 to 0.17; I^2^: from 95% to 0%), and T levels (pooled effect size: from 8.71 to 3.73; I^2^: from 92% to 0%). These findings collectively indicate that this study had a profound influence on multiple outcomes.

### Primary outcomes: effects of vitamin d supplementation on semen parameters and live birth rate

#### Semen volume

**Figure 2:**
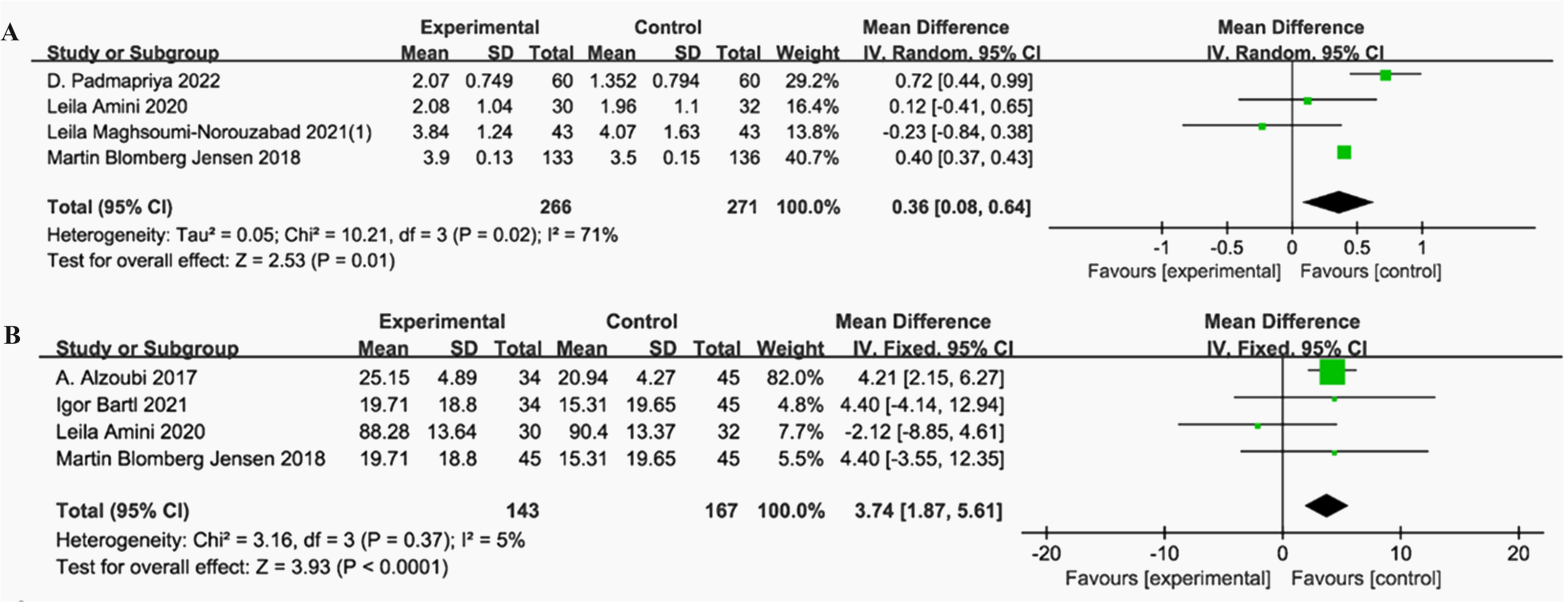
Forest plot of the meta-analysis examining the effect of vitamin D supplementation on semen volume and sperm concentration. (A) Semen volume. (B) Sperm concentration.

Our meta-analysis (*n* = 537) demonstrated a statistically significant increase in semen volume following vitamin D supplementation (MD = 0.36 mL, *P* = 0.01) (Amini et al., 2020; Blomberg Jensen et al., 2018; Maghsoumi-Norouzabad et al., 2021; Padmapriya et al., 2022). The study by Blomberg Jensen et al. (2018), contributing 40.7% of the weight, showed the most precise effect estimate (Blomberg Jensen et al., 2018). Considerable heterogeneity was observed across studies (I^2^ = 71%, *P* = 0.02) (Fig. 2A).

#### Sperm concentration

The meta-analysis (*n* = 310) revealed a statistically significant increase in sperm concentration (×10^6^/mL) after vitamin D supplementation (MD=3.74, *P* < 0.0001) (Alzoubi et al., 2017; Amini et al., 2020; Bartl et al., 2021; Blomberg Jensen et al., 2018). Heterogeneity was low (I^2^ = 5%, *P* = 0.37), with Alzoubi et al. (2017) contributed 82% of the weight (Fig. 2B).

#### Progressive sperm motility

**Figure 3:**
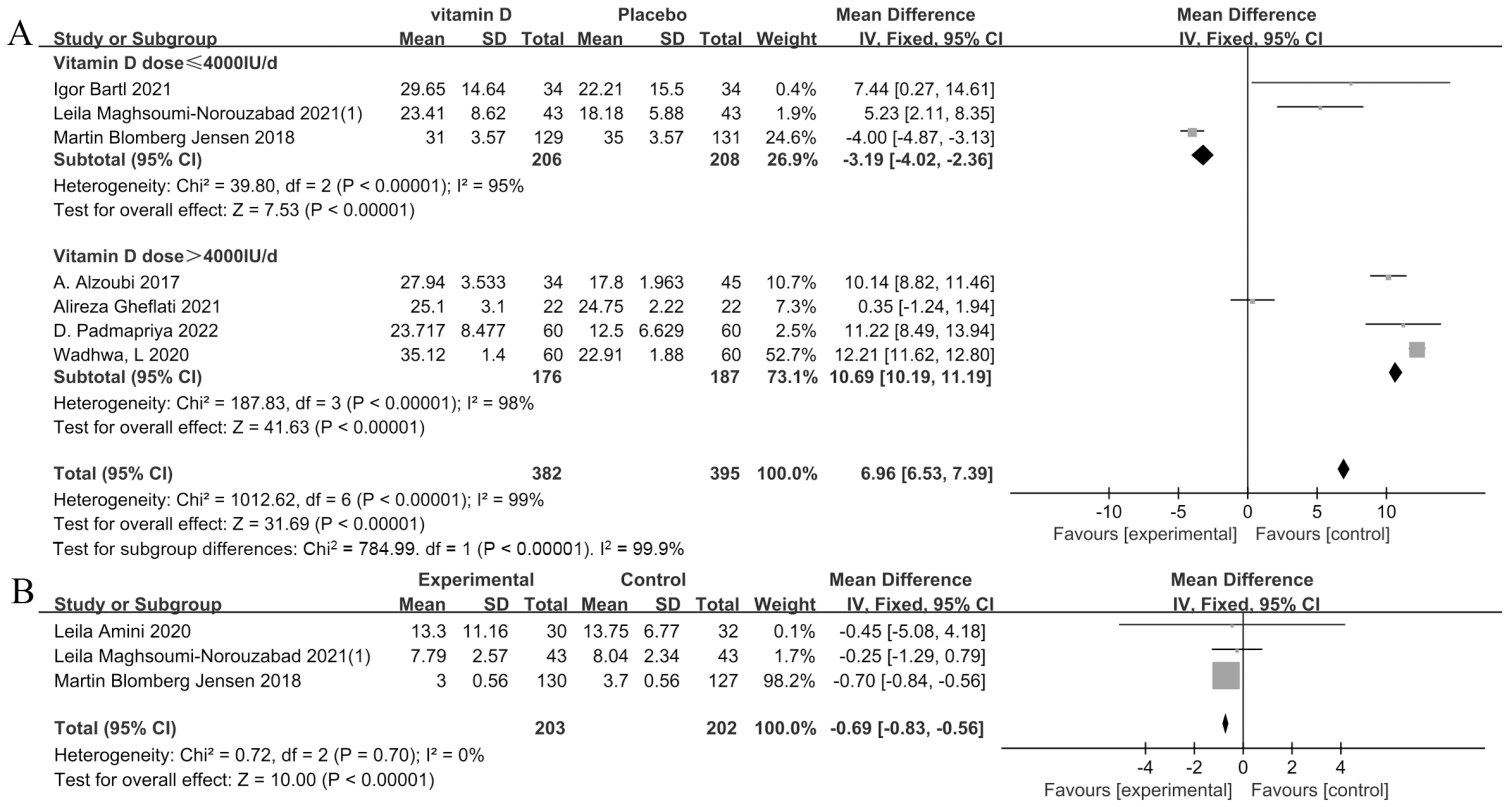
Forest plot of the meta-analysis examining the effect of vitamin D supplementation on progressive sperm motility and normal sperm morphology. (A) Progressive sperm motility. (B) Normal sperm morphology.

The meta-analysis (*n* = 777) revealed a statistically significant increase in progressive sperm motility (%) after vitamin D supplementation (MD = 6.96, *P* < 0.00001) (Alzoubi et al., 2017; Bartl et al., 2021; Blomberg Jensen et al., 2018; Gheflati et al., 2021; Maghsoumi-Norouzabad et al., 2021; Padmapriya et al., 2022; Wadhwa et al., 2020). The subgroup analysis by vitamin D dosage (≤4,000 IU/d *vs.* > 4,000 IU/d) did not effectively reduce heterogeneity, suggesting the presence of potential covariates influencing the outcomes. Therefore, a meta-regression was conducted to further explore these sources of variability (Fig. 3A).

#### Normal sperm morphology

The meta-analysis of (*n* = 405) normal sperm morphology revealed a statistically significant overall decrease with vitamin D supplementation (MD = −0.69, *P* < 0.00001) (Amini et al., 2020; Blomberg Jensen et al., 2018; Maghsoumi-Norouzabad et al., 2021). However, this finding likely reflects a baseline imbalance rather than a true adverse effect of vitamin D. The study by Blomberg Jensen et al. (2018), contributing 98.2% of the weight, showed the most precise effect estimate. Low heterogeneity was observed across studies (I^2^ = 0%, *P* = 0.7) (Fig. 3B).

#### Clinical pregnancy rate and live birth rate

The meta-analysis included two studies evaluating the impact of vitamin D supplementation on clinical pregnancy rates (Banks et al., 2021; Wadhwa et al., 2020). The pooled analysis demonstrated an increased risk ratio (RR) of 2.20 (*p* = 0.61), indicating no statistically significant difference between the vitamin D supplementation and control groups. However, the heterogeneity was considerable (I^2^ = 78%, *p* = 0.03), suggesting substantial variability between the studies.

#### Live birth rate

**Figure 4:**
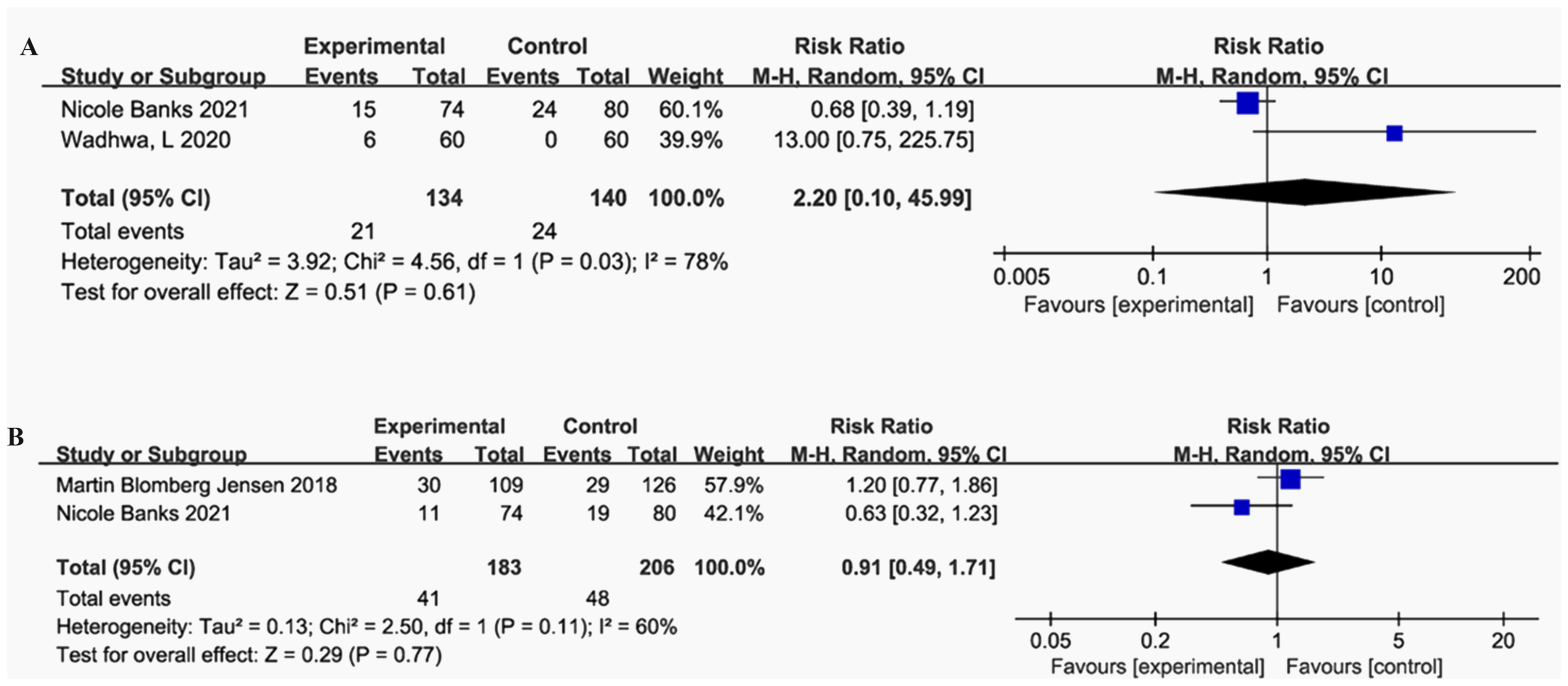
Forest plot of the meta-analysis examining the effect of vitamin D supplementation on clinical outcomes. (A) Clinical pregnancy rate. (B) Live birth rate.

The pooled RR increase for live birth rate after vitamin D supplementation was 0.91 (*P* = 0.77), indicating no significant effect of vitamin D supplementation (Banks et al., 2021; Blomberg Jensen et al., 2018). Moderate heterogeneity was observed across studies (I^2^ = 60%, *P* = 0.11) (Figs. 4A–4B).

Taken together, vitamin D supplementation showed significant improvements in semen volume, sperm concentration, and progressive sperm motility, while no significant effects were observed on clinical pregnancy or live birth rates in this study.

### Secondary outcomes: effects of vitamin D supplementation on hormonal and reproductive parameters

#### FSH levels

**Figure 5:**
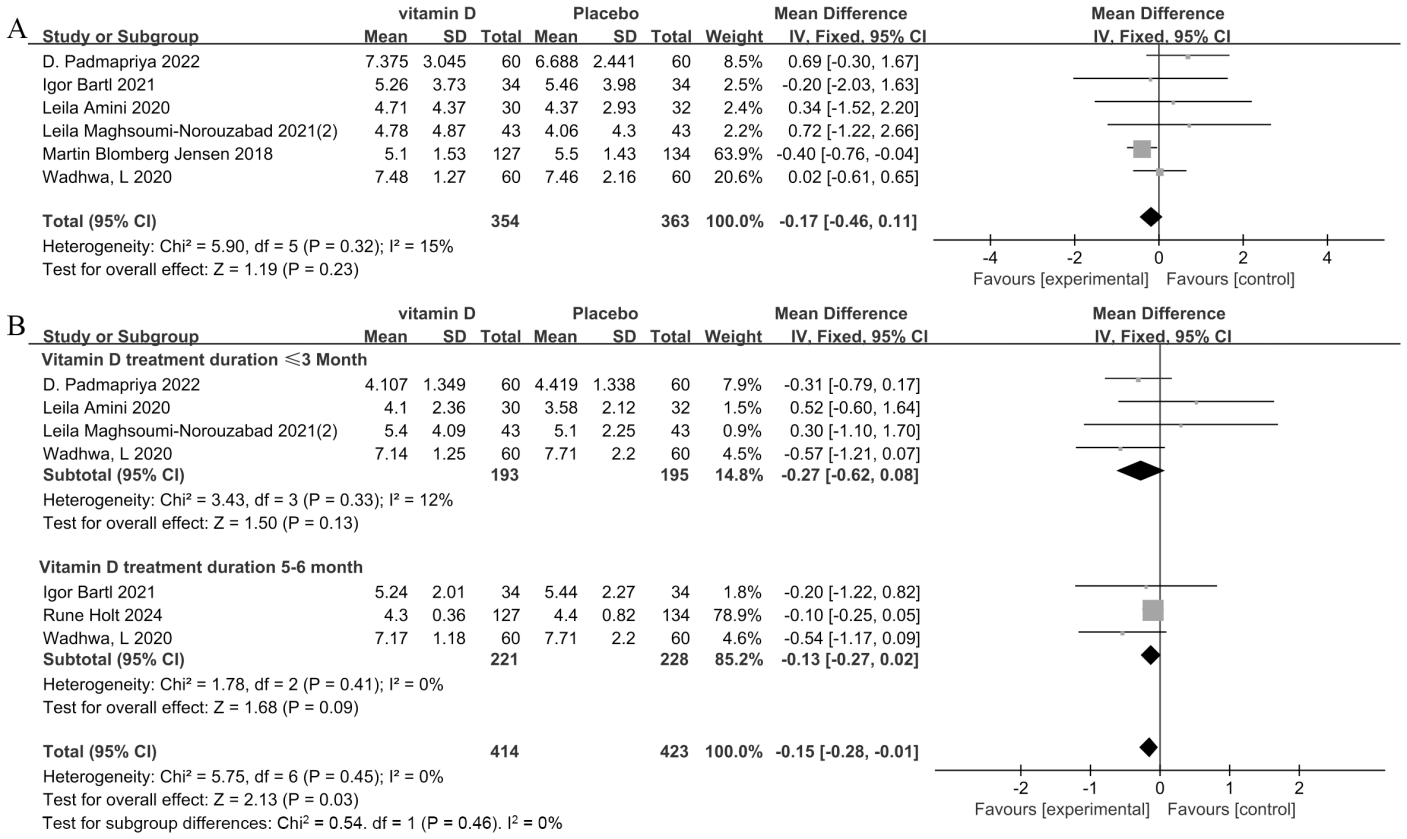
Forest plot of the meta-analysis examining the effect of vitamin D supplementation on FSH and LH in infertile men. (A) FSH. (B) LH.

The meta-analysis (*n* = 717 participants) showed no significant effect of vitamin D supplementation on FSH levels (MD = −0.17 IU/L, *P* = 0.23) (Amini et al., 2020; Bartl et al., 2021; Blomberg Jensen et al., 2018; Maghsoumi-Norouzabad et al., 2021; Padmapriya et al., 2022; Wadhwa et al., 2020). Heterogeneity was low (I^2^ = 15%, *P* = 0.32), with Blomberg Jensen et al. (2018) contributing 63.9% of the weight (Fig. 5A). Individual study estimates consistently demonstrated null effects, with all confidence intervals overlapping zero. Subgroup analysis by treatment duration (*P* = 0.06) did not reach statistical significance, though effects differed numerically between 5–6-month (MD = −0.29 IU/L) and ≤3-month (MD=0.28 IU/L) interventions (Fig. S4).

#### LH levels

The meta-analysis (*n* = 837 participants) demonstrated a borderline significant reduction in LH levels with intervention (MD = −0.15 IU/L, *P* = 0.03), with no observed heterogeneity (I^2^ = 0%, *P* = 0.45) (Amini et al., 2020; Bartl et al., 2021; Holt et al., 2024; Maghsoumi-Norouzabad et al., 2021; Padmapriya et al., 2022; Wadhwa et al., 2020). The effect was largely driven by Holt et al. (2024), which contributed 89.1% of the weight. Subgroup analysis by treatment duration (*P* = 0.21) did not reach statistical significance. While the overall effect approached significance, the confidence interval marginally crossed the null value (Fig. 5B).

#### SHBG levels

**Figure 6:**
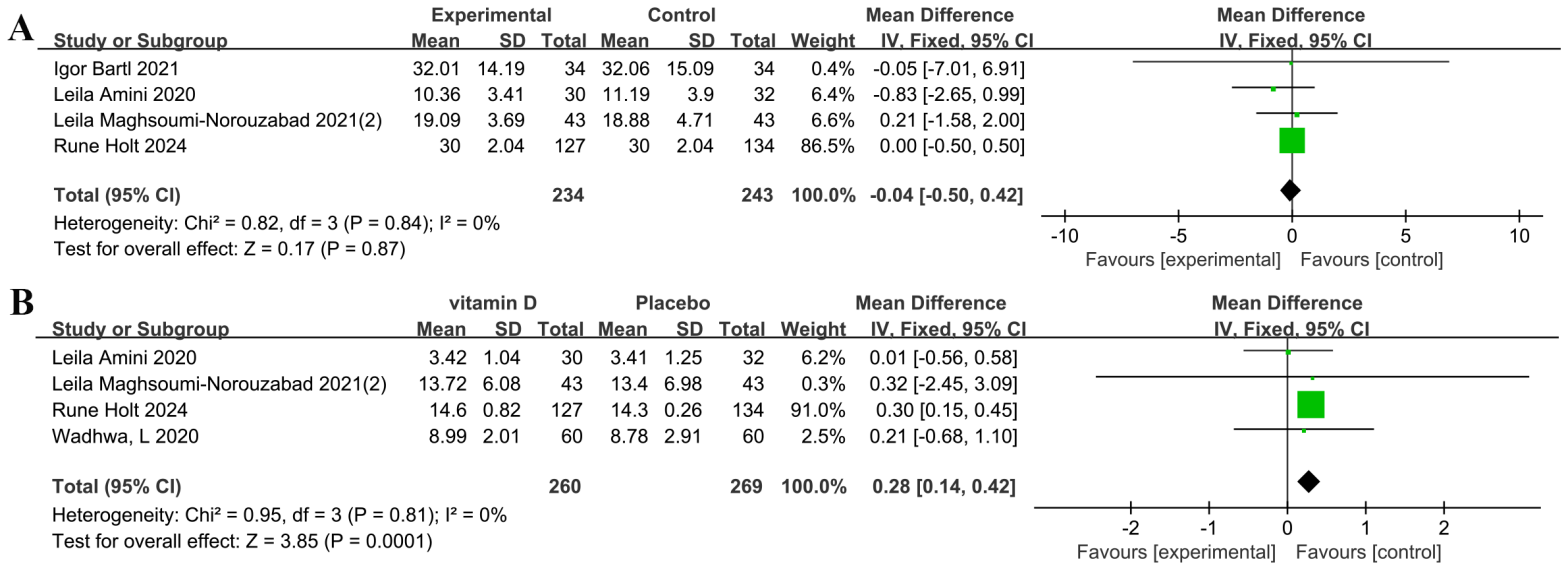
Forest plot of the meta-analysis examining the effect of vitamin D supplementation on SHBG and T in infertile men. (A) SHBG. (B) T.

The meta-analysis of four studies (*n* = 477) revealed no significant effect of vitamin D supplementation on SHBG levels (MD = −0.04 nmol/L, *P* = 0.87), with perfect consistency across studies (I^2^ = 0%) (Amini et al., 2020; Bartl et al., 2021; Holt et al., 2024; Maghsoumi-Norouzabad et al., 2021) (Fig. 6A).

#### T levels

The meta-analysis demonstrated a highly significant overall effect of vitamin D supplementation on T levels (MD: 0.28 nmol/L, *P* = 0.0001) (Amini et al., 2020; Holt et al., 2024; Maghsoumi-Norouzabad et al., 2021; Wadhwa et al., 2020). Substantial heterogeneity was observed across studies (I^2^ = 0%, *P* = 0.81) (Fig. 6B).

Overall, this study found that vitamin D supplementation was associated with a significant increase in serum T levels, had minimal effects on LH, and showed no observable effects on FSH or SHBG levels.

### Meta-regression

Given the high consistency across merged datasets, meta-regression analyses were conducted to evaluate the effects of vitamin D supplementation on semen volume and progressive sperm motility in infertile men, adjusting for baseline covariates.

For semen volume, both frequentist and Bayesian models indicated that higher BMI might be associated with lower semen volume, while higher vitamin D levels were associated with slightly higher volume. However, these trends were weak and did not reach statistical significance.

In contrast, for sperm motility, the Bayesian model suggested a stronger positive association with baseline vitamin D levels, indicating that men with higher vitamin D may experience more pronounced improvement in motility. The effect of BMI remained inconsistent across models.

These findings highlight the potential for personalized treatment considerations. Full parameter estimates and convergence diagnostics are presented in Supplementary materials (Table S1 and Figs. S5–S6).

## Discussion

### Key findings

This systematic review and meta-analysis suggest that vitamin D supplementation modestly improves semen volume (+0.36 mL), sperm concentration (+3.74 ×10^6^/mL), and progressive motility (+6.96%) in infertile men. Additionally, T levels increased significantly (+0.28 nmol/L), while LH levels declined marginally (−0.15 IU/L). However, no significant changes were observed in clinical pregnancy or live birth rates. These results suggest that vitamin D may positively influence spermatogenesis and hormonal function, though current evidence remains insufficient to confirm effects on fertility endpoints.

### Interpretation of unexpected findings

A statistically significant reduction in normal sperm morphology was detected (MD = −0.69%). Upon closer examination, this result may be attributed to baseline imbalances. In the study by Blomberg Jensen et al. (2018), which contributed 98.2% of the pooled weight, the control group exhibited a substantially higher mean baseline morphology compared to the intervention group (3.8 ± 3.1% *vs.* 2.9 ± 2.6%). This disproportion may have skewed the post-intervention comparison. Therefore, the observed reduction likely reflects methodological limitations rather than the true adverse effect of vitamin D.

### Sources of heterogeneity and meta-regression insights

To address between-study heterogeneity, we conducted subgroup analyses and sensitivity analyses focusing on dosing regimens, baseline vitamin D status, and outcome definitions, which collectively indicated that baseline vitamin D status and population characteristics were the primary contributors to heterogeneity.

Compared to the extensive research on female reproductive health, studies investigating the effects of vitamin D supplementation on clinical outcomes in infertile men remain limited. In our meta-analysis, only two trials reported clinical pregnancy rates (Banks et al., 2021; Wadhwa et al., 2020), and no statistically significant benefit was observed, even after subgroup analyses stratified by baseline 25(OH)D levels (Fig. S7).

Despite subgrouping by dose and duration, several included trials administered vitamin D together with calcium, which may have contributed to heterogeneity and complicate attribution of the observed effects to vitamin D alone. The limited number of such trials precluded robust subgroup analyses. Sensitivity analysis further indicated that exclusion of trials with calcium co-supplementation did not materially reduce heterogeneity in outcomes with substantial variability, suggesting that factors beyond calcium co-supplementation may have contributed to the observed heterogeneity.

Although the study by Gheflati et al. (2021) was rated as low risk of bias and reported multiple outcomes, it introduced considerable heterogeneity in several pooled analyses. We therefore excluded it from selected sensitivity analyses and retained only its data on progressive sperm motility. This influence may be attributed to three factors: a small sample size (*n* = 22 in the intervention group), inclusion of only asthenozoospermia patients, and use of a high-dose intermittent regimen (50,000 IU/week), which differed from the daily dosing applied in most other studies.

In meta-regression models for semen volume, the frequentist analysis identified a marginally significant inverse association with BMI, whereas the Bayesian model did not confirm this finding. Conversely, in the analysis of progressive sperm motility, Bayesian regression detected a significant positive association with baseline 25(OH)D levels, which was not observed in the frequentist model. These discrepancies may reflect differences in how the two frameworks handle small sample sizes and uncertainty. Given this context, we prioritized Bayesian results where model disagreement occurred, particularly as they offer a probabilistic interpretation more suited to limited data and clinically complex outcomes.

### Clinical implications

Our meta-analysis demonstrated a statistically significant increase in semen volume by 0.36 mL with vitamin D supplementation, this change corresponds to approximately 24% of the WHO-defined threshold for hypospermia (1.5 mL) (Björndahl & Kirkman Brown, 2022). Increasing semen volume may improve fertility by boosting total sperm count, aiding cervical mucus penetration, and improving sperm delivery (Katz, Overstreet & Hanson, 1980). These benefits are especially important for men with borderline low semen volume, where even modest increases may enhance natural conception chances.

Concurrently, sperm concentration rose by 3.74 ×10^6^/mL, equivalent to 25% of the WHO reference limit (15 ×10^6^/mL). For men with mild oligozoospermia, this could shift them into the normal range. Previous studies have shown that low sperm concentration adversely affects natural conception rates (Bonde et al., 1998), while even moderate increases in sperm concentration are significantly associated with shorter time to pregnancy (Romero Herrera et al., 2021). These findings highlight the potential clinical relevance of our results in improving male reproductive potential (Romero Herrera et al., 2021).

Most notably, total motile sperm count is widely recognized as a more effective predictor of male fertility compared to conventional individual parameters (Hamilton et al., 2015). In our study, progressive sperm motility improved by 6.96% (exceeding the proposed minimal clinically important difference (MCID) of 5%), which is clinically meaningful given that even 5% increases in progressive sperm motility are associated with higher natural pregnancy rates (Esteves, 2014). This enhancement may contribute to improved fertility by promoting more efficient sperm migration through the female reproductive tract (Marchlewska et al., 2024) and reflecting better mitochondrial function and energy metabolism (Amaral, Ramalho-Santos & John, 2007)—both essential for successful fertilization.

However, our study did not find any effective influence of vitamin D supplementation on clinical pregnancy and live birth outcomes, and the data from the few available studies exhibited high heterogeneity. Therefore, the present findings do not support a conclusive benefit of vitamin D supplementation on fertility outcomes beyond the improvements observed in semen parameters.

Our meta-analysis revealed a statistically significant elevation in serum T levels following vitamin D supplementation (0.28 nmol/L). Although this magnitude of increase falls below the proposed clinically important difference of 1.7 nmol/L (Travison et al., 2017), it represents a significant improvement from participant baseline values (range: 3–15 nmol/L) recruited in our study. Notably, these findings were accompanied by a subtle yet statistically significant reduction in LH levels (−0.15 IU/L). The inverse relationship may reflect vitamin D-mediated augmentation of Leydig cell sensitivity.

While no significant changes were observed in FSH or SHBG levels, this does not necessarily imply a lack of effect but rather reflects current limitations in evidence and guidance. Importantly, our findings underscore the lack of clinical justification for exceeding the recommended daily dose of vitamin D (≤4,000 IU/day) in infertile men. Clinicians should prioritize adherence to established dosing thresholds while monitoring baseline 25(OH)D levels to optimize therapeutic efficacy and safety.

Although vitamin D synthesized through sunlight may have certain biological advantages, studies have shown that both sun exposure and oral supplementation effectively increase serum 25(OH)D levels, with oral supplementation often proving more efficient (Joh et al., 2020; Mo et al., 2025; Wicherts et al., 2011). Given the variability in sunlight exposure due to factors such as latitude, season, and lifestyle, cutaneous synthesis tends to be inconsistent (Giustina et al., 2024). Therefore, oral supplementation remains a more reliable and practical approach for clinical management, particularly in infertile men.

Furthermore, this meta-analysis focused exclusively on oral vitamin D supplementation. While this route is common in clinical settings (Giustina et al., 2024), previous studies have suggested that vitamin D synthesized through sunlight exposure may differ in bioavailability or physiological effects. As non-supplemental sources were not evaluated in the included studies, the findings presented here may not fully represent the effects of vitamin D obtained through natural means.

### Interpretation of clinical outcomes

In our study, no significant improvement in clinical pregnancy rates was observed with vitamin D supplementation. However, the lack of a significant effect should be interpreted with caution, given several contextual factors. First, both trials had relatively small sample sizes and were likely underpowered to detect meaningful differences in clinical pregnancy (*n* = 134) or live birth outcomes (*n* = 183). Second, the pooled data on live birth rates demonstrated substantial heterogeneity, which may stem from marked differences in participant characteristics as well as the lack of standardized outcome definitions across studies (Banks et al., 2021; Blomberg Jensen et al., 2018). Specifically, one study enrolled a heterogeneous population in which only a minority (26 out of 154) were vitamin D-deficient (Banks et al., 2021), whereas the other exclusively included vitamin D-deficient men (*n* = 235, mean level: 35 ± 11 nmol/L) (Blomberg Jensen et al., 2018). These baseline differences likely contributed to the variability in effect estimates and highlight the need for better-defined inclusion criteria in future trials.

Notably, semen quality is not an optimal predictor of male fertility potential but rather a soft endpoint, it remains in clinical use due to the lack of better alternatives (Blomberg Jensen et al., 2018; Guzick et al., 2001; Lamb & Marinaro, 2023; Zinaman et al., 2000). Therefore, improvements in semen parameters do not imply a direct causal relationship with clinical reproductive outcomes. Moreover, pregnancy outcomes are influenced not only by male factors but also by female conditions (Jiang et al., 2025). Factors such as maternal age, BMI, ovary reserve, uterine receptivity, endocrine status, and prior reproductive history have been shown to substantially affect clinical pregnancy and live birth outcomes (Ata et al., 2023; Caldwell et al., 2024; Carson & Kallen, 2021; Li et al., 2024; Sabbagh et al., 2025), potentially modifying or obscuring the effects of male-focused interventions.

Hence, the potential benefits of vitamin D for reproductive outcomes may require further verification through larger sample sizes or more precise population stratification studies. Additionally, in line with recent international consensus, pregnancy and live birth, beyond semen analysis, should receive greater attention as core clinical endpoints reflecting true reproductive outcome (Rimmer et al., 2025).

### Mechanistic considerations

Vitamin D supplements are activated through a series of transformations after absorption (Giustina et al., 2024). Once activated, vitamin D seeks out select cells and organs that have a sensor known as VDR (Evans & Lippman, 2020). The VDR and its metabolic enzymes are concomitantly distributed throughout the male reproductive system (Blomberg Jensen, 2014). Consistent with this, transcriptomic data from the Mammalian Reproductive Genetics Database V2 (MRGD V2) demonstrate that VDR is expressed across multiple testicular cell types, including spermatogonia, spermatocytes, and Sertoli cells (Fig. S8) (Jelinsky et al., 2007; Johnston et al., 2005; Johnston et al., 2008; Robertson et al., 2020), supporting its potential regulatory role in male reproductive function. This characteristic suggests the testis may autonomously regulate local vitamin D activity, highlighting its potential regulatory role in male reproductive function.

From an epigenetic viewpoint, a systematic review concluded that aberrant DNA methylation in human sperm was associated with male infertility and abnormal semen parameters (Asenius, Danson & Marzi, 2020). Recent studies provided direct evidence that infertile men exhibit significantly elevated methylation levels in the promoter region of the VDR gene in both peripheral blood and semen samples (Hussein et al., 2021; Vladoiu et al., 2017). Notably, this aberrant methylation pattern shows a negative correlation with serum 1,25(OH)_2_D_3_ concentrations (Hussein et al., 2021; Vladoiu et al., 2017). Given the role of vitamin D deficiency, it was reasonable to speculate that supplementation of vitamin D may ameliorate male infertility.

Vitamin D exerts antioxidant and anti-inflammatory effects through upregulation of GPX4, SOD, and suppression of NF-κB signaling, which may improve the testicular microenvironment (Jueraitetibaike et al., 2019). The optimized microenvironment is crucial for normal spermatogenesis and T synthesis. The binding of 1,25(OH)_2_D_3_ to VDR modulates key steroidogenic enzymes, enhancing T biosynthesis (Hu et al., 2023), which is crucial for spermatogenesis. In addition, vitamin D exerts protective effects on testicular spermatogenic cells and Sertoli cells by inhibiting apoptosis in both cell types (Chu, Deng & Fang, 2024). Collectively, these mechanisms delineate the molecular basis of vitamin D’s regulatory role in testicular function.

*In vitro* experiments have revealed that vitamin D increased intracellular calcium from an intracellular calcium storage in the neck of human spermatozoa, induced sperm motility, and the acrosome reaction (Blomberg Jensen et al., 2011). Moreover, vitamin D was demonstrated to stimulate the expression of VDR in a dose-dependent manner at the cellular level (Nangia et al., 2007). Animal studies have further demonstrated that vitamin D enhances the expression of the calcium-binding protein Calbindin-D28k (Boisen et al., 2021), which is critical for maintaining calcium ion homeostasis in the seminiferous tubules. This mechanism supports sperm flagellar motility, consistent with the observed improvement in progressive sperm motility in our study.

### Strengths and limitations

To our knowledge, this is the first meta-analysis to jointly evaluate semen quality, reproductive hormone and fertility outcomes in infertile men receiving vitamin D supplementation.

Notably, the limited reporting of clinical pregnancy and live birth outcomes, coupled with high heterogeneity in the available data, restricts our ability to draw definitive conclusions about the clinical efficacy of vitamin D supplementation. A formal GRADE assessment was not performed since the number of studies included was limited. Additionally, although several hormonal changes were statistically significant, their clinical relevance remains uncertain, as these effects may not meet established thresholds for meaningful improvement.

None of the included studies reported any adverse events related to vitamin D supplementation. However, since most trials did not explicitly describe adverse event monitoring procedures or predefined safety outcomes, and therefore the absence of reported events should be interpreted with caution. Further studies with longer follow-up periods, larger sample sizes, and standardized definitions of MCID are essential to clarify the impact of vitamin D supplementation on clinical fertility outcomes.

## Conclusions

Vitamin D supplementation was found to modestly improve semen volume, sperm concentration, progressive sperm motility, and T levels in infertile men. Notably, the improvement in progressive sperm motility (+6.96%) exceeded the proposed MCID, which is considered predictive of higher natural pregnancy rates. Importantly, the positive association between vitamin D supplementation and baseline 25(OH)D levels was significant only in the Bayesian analysis and should be interpreted with caution. Overall, vitamin D supplementation may be most beneficial for men with confirmed deficiency, while routine use without prior testing shows limited evidence of benefit. Higher-than-recommended doses (>4,000 IU/day) did not provide additional clinical benefit.

Due to the limited number of included studies and variations in baseline characteristics across study populations, no beneficial effect of vitamin D supplementation was observed on clinical pregnancy or live birth rates. Meta-regression further suggested that baseline vitamin D levels and BMI may act as potential effect modifiers.

We recommend that clinicians assess vitamin D status in infertile men and consider supplementation within established safety thresholds when deficiency is identified. Future studies should focus on evaluating the impact of vitamin D on male reproductive health with longer follow-up periods to determine its effects on clinical fertility outcomes. Large-scale trials stratified by baseline vitamin D status and BMI are also warranted to clarify the dose–response relationship and reduce heterogeneity in observed effects.

##  Supplemental Information

10.7717/peerj.21002/supp-1 Supplemental Information 1 PRISMA abstract checklist

10.7717/peerj.21002/supp-2 Supplemental Information 2 PRISMA Checklist

10.7717/peerj.21002/supp-3 Supplemental Information 3 Data extracted from recruited article

10.7717/peerj.21002/supp-4 Supplemental Information 4 Supplementary Materials

10.7717/peerj.21002/supp-5 Supplemental Information 5 Vitamin D dose and intervention duration across studies. Blue bars represent the estimated daily dose (IU/day) of vitamin D supplementation, and grey bars represent the intervention duration (weeks)

10.7717/peerj.21002/supp-6 Supplemental Information 6 Summary of risk of bias assessments for included studies (A) ROB2 was applied for RCTs, and (B) ROBINS-I for observational studies.

10.7717/peerj.21002/supp-7 Supplemental Information 7 Subgroup analysis of semen volume and progressive sperm motility (A) Semen volume by intervention dosage. (B) Progressive sperm motility by intervention duration.

10.7717/peerj.21002/supp-8 Supplemental Information 8 Subgroup analysis of FSH levels by intervention duration Subgroup analysis of FSH levels by treatment duration showed a borderline significant interaction (P = 0.06), with numerically divergent effects between longer (5–6 months, MD = –0.29 IU/L) and shorter (≤3 months, MD = 0.28 IU/L) interventions (P=0.06) . This finding suggests a potential benefit of longer-term vitamin D supplementation on FSH regulation.

10.7717/peerj.21002/supp-9 Supplemental Information 9 Meta-regression of semen volume based on baseline of 25(OH)D (ng/ml) and BMI (Kg/m 2 ) The traditional weighted linear regression analysis suggested that baseline 25(OH)D levels might be positively associated with semen volume, while BMI could be negatively associated .

10.7717/peerj.21002/supp-10 Supplemental Information 10 Meta-regression of progressive sperm motility based on baseline of 25(OH)D (ng/ml) and BMI (Kg/m 2 ) T he Bayesian meta-regression analysis showed a significant positive association between baseline 25(OH)D levels and sperm motility, while the traditional method did not detect such an association.

10.7717/peerj.21002/supp-11 Supplemental Information 11 Subgroup analysis of clinical pregnancy rates by baseline vitamin D status S ubgroup analyses stratified by participants’ baseline VD levels demonstrated no statistically significant effect of VD supplementation on clinical pregnancy rates .

10.7717/peerj.21002/supp-12 Supplemental Information 12 Expression of VDR , CYP27B1, and CYP2R1 in human testicular and epididymal tissues and cells . Notes: VDR ( ENSMUSG00000111424 ), CYP27B1 ( ENSMUSG00000111012 ), CYP2R1 ( ENSMUSG00000186104 ) RNA-seq data from the * MRGD V2* demonstrate that the VDR , CYP27B1, and CYP2R1A are expressed across multiple human testicular cell populations. Detectable VDR transcripts are observed in spermatogonia (SSEA4, KIT, and undifferentiated subtypes), spermatocytes, and Sertoli cells, indicating both germ-cell and somatic expression. In addition, expression s are also seen in the epididy mi s (caput, corpus, and cauda). These results provide molecular evidence that vitamin D signaling components are locally present in the male reproductive tract, supporting a potential role for VDR-mediated pathways in spermatogenesis and sperm maturation.

10.7717/peerj.21002/supp-13 Supplemental Information 13 Evaluation of the effects of vitamin D supplementation on semen parameters and reproductive hormones in infertile men the findings indicated improvements in semen volume, sperm concentration, progressive sperm motility, and testosterone levels, suggesting a potential role of vitamin D supplementation in male infertility management.

## Additional Information and Declarations

### Data Availability

The following information was supplied regarding data availability: This is a systematic review and meta-analysis.
