# Supplementary material for: Vitamin D supplementation in infertile men: a systematic review and meta-analysis of effects on semen quality and endocrine function
Source: PeerJ. 2026 Apr 13;14:e21002. doi: 10.7717/peerj.21002 (PMC13086023; doi:10.7717/peerj.21002)
Supplement: Supplemental Information 4 [file peerj-14-21002-s004.docx]

**Supplementary Methods**

**Frequentist and Bayesian and Meta-Regression**

To explore sources of heterogeneity not accounted for by subgroup or sensitivity analyses, we performed both frequentist and Bayesian meta-regressions. These models aimed to assess the modifying effects of baseline vitamin D status [25(OH)D] and body mass index (BMI) on the outcomes of vitamin D supplementation

**Frequentist Meta-Regression**

We first applied weighted linear regression models using the metafor package in R (version 4.3.1). Inverse-variance weights were calculated based on the combined sample sizes of the intervention and control groups. Covariates (25(OH)D and BMI) were standardized (z-scores) to allow direct comparison of effect sizes. Studies with missing covariate data were excluded from the meta-regression.

**Bayesian Meta-Regression**

To complement the frequentist results and allow probabilistic interpretation, Bayesian meta-regression was conducted using the brms package. Models were fitted assuming a Gaussian family distribution with identity link functions. Weakly informative priors were used by default unless otherwise stated.

Markov Chain Monte Carlo (MCMC) simulations were run using Stan backend with 2 chains, each with 2,000 iterations (1,000 warm-up). Convergence was assessed using Rhat (<1.01 deemed acceptable) and effective sample sizes (ESS > 400). Divergent transitions (n=2) were addressed by increasing adapt_delta to 0.99. Posterior distributions were summarized using posterior means and 95% credible intervals (CrI). These Bayesian outputs were interpreted in conjunction with the frequentist findings to enhance robustness.

**Supplementary Results**

**Study and participant characteristics**

**
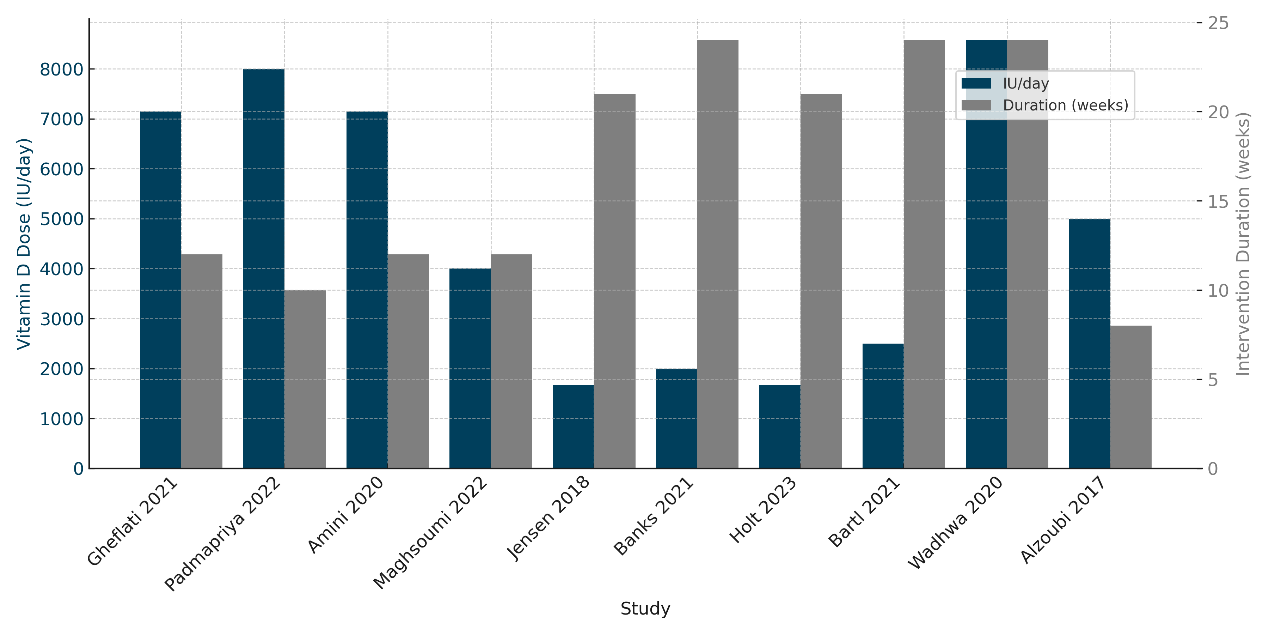
**

**Figure S1.** Vitamin D dose and intervention duration across studies.

Blue bars represent the estimated daily dose (IU/day) of vitamin D supplementation, and grey bars represent the intervention duration (weeks).

**Risk of Bias**


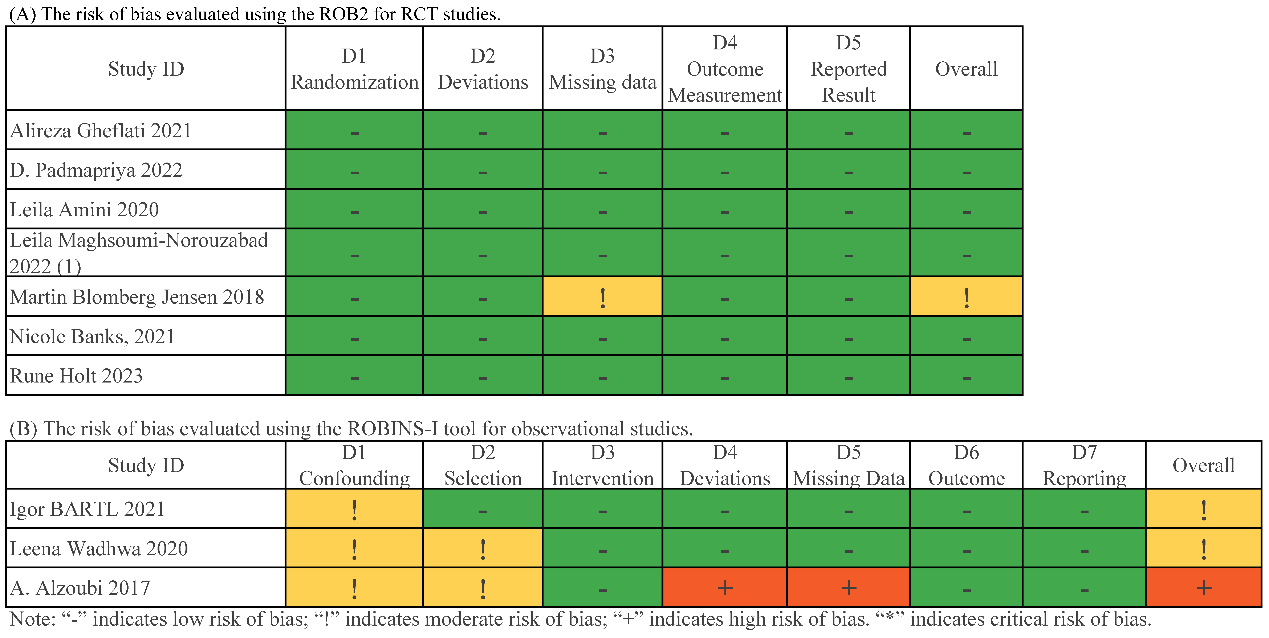


Figure S2. Summary of risk of bias assessments for included studies. (A) ROB2 was

applied for RCTs, and (B) ROBINS-I for observational studies.

The risk of bias was assessed using the Cochrane Risk of Bias 2 (ROB 2) tool. Each domain is categorized as “low risk,” “some concerns,” or “high risk” based on the methodological quality of included studies.

**Subgroup analysis**


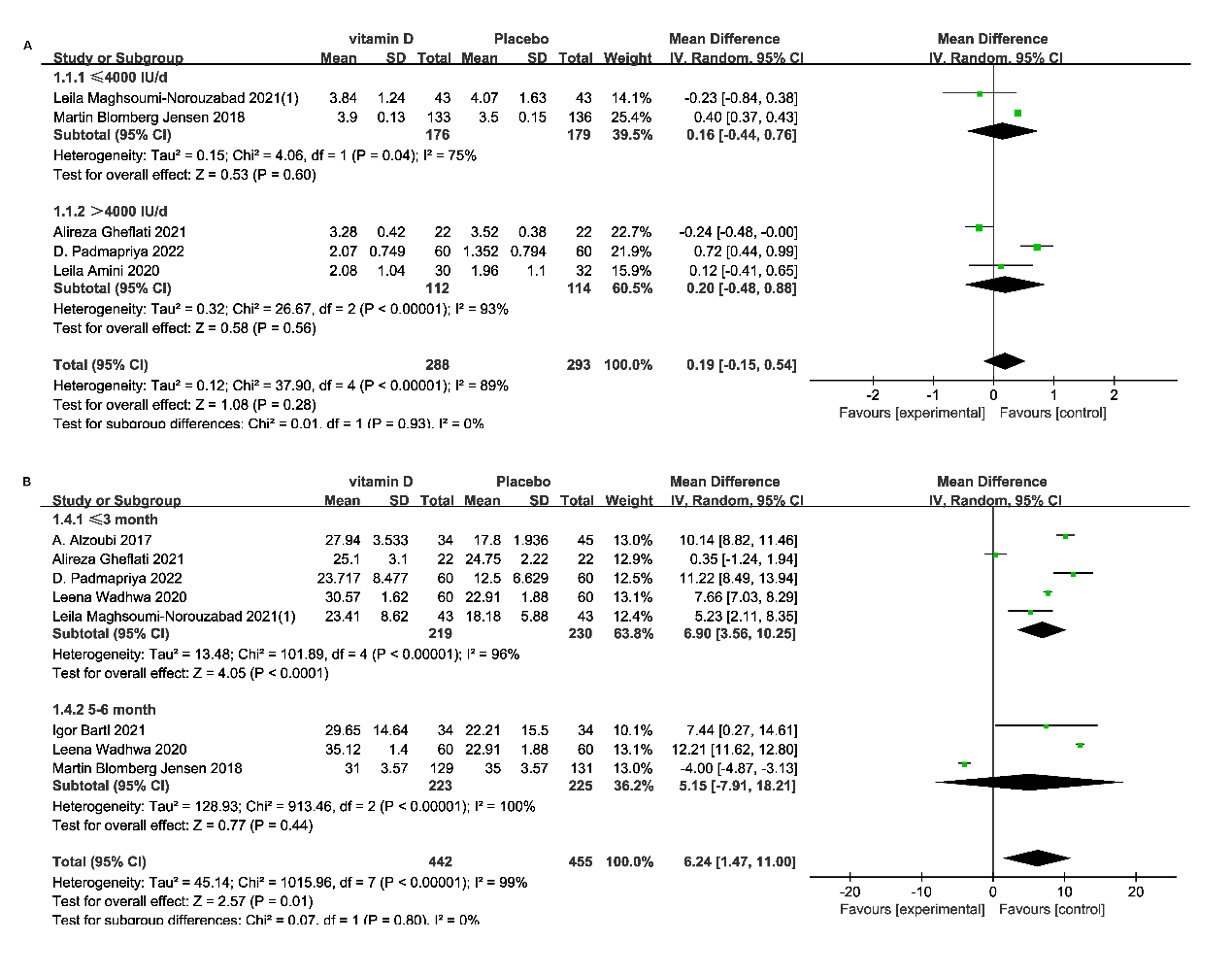


Figure S3. Subgroup analysis of semen volume and progressive sperm motility. (A) Semen volume by intervention dosage. (B) Progressive sperm motility by intervention duration.

To explore the substantial heterogeneity observed in the pooled analyses, we conducted subgroup analyses based on intervention dose and duration for semen volume (Figure S2A) and progressive sperm motility (Figure S2B). However, these subgroupings did not fully account for the heterogeneity. In the semen volume analysis, high-dose supplementation (>4000 IU/day) did not show additional benefit (MD = 0.15 mL, 95% CI: –0.08 to 0.38). Considering progressive motility, although differences were observed between dose-based subgroups, heterogeneity remained considerable, likely due to residual confounding factors (Figure 4C).


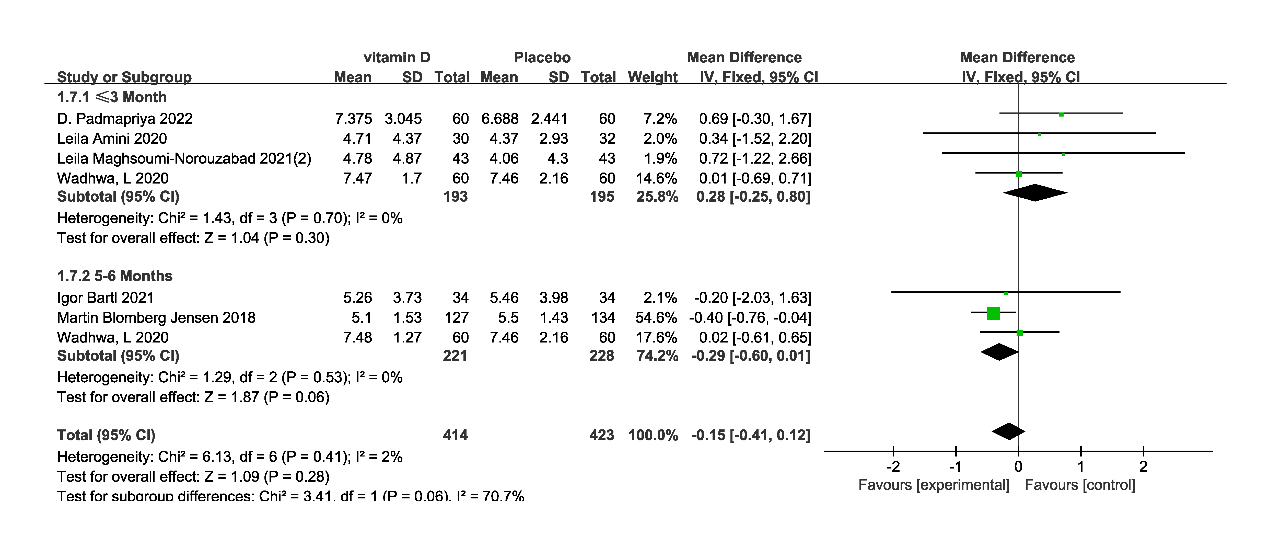


Figure S4. Subgroup analysis of FSH levels by intervention duration.

Subgroup analysis of FSH levels by treatment duration showed a borderline significant interaction (P = 0.06), with numerically divergent effects between longer (5–6 months, MD = –0.29 IU/L) and shorter (≤3 months, MD = 0.28 IU/L) interventions (P=0.06). This finding suggests a potential benefit of longer-term vitamin D supplementation on FSH regulation.

**Meta regression**

**Table 3.** Effects of baseline 25(OH)D and BMI on semen parameters: Dual regression analyses of volume and progressive sperm motility.

| Semen volume (n=4) (a) Weighted linear regression. (b) Bayesian meta-regression | | | | | | | Residual SE= 0.722 (df = 1) Adjusted R² = 0.956 F-statistic = 33.52 (df = 2,1), p = 0.121 |
| --- | --- | --- | --- | --- | --- | --- | --- |
| (a) | Variable | Coefficient (β) | SE | 95% CI | t-value | p-value |  |
|  | Intercept | 2.528 | 0.483 | (-1.569, 6.625) | 5.231 | 0.12 |  |
|  | Baseline 25(OH)D (ng/ml) | 0.028 | 0.01 | (-0.014, 0.068) | 2.882 | 0.213 |  |
|  | BMI (kg/m²) | -0.116 | 0.017 | (-0.188, -0.043) | -6.823 | 0.093† |  |
| (b) | Variable | Posterior Mean | Posterior SD | 95% CI | Rhat | Bulk ESS | Tail ESS |
|  | Intercept | 0.03 | 0.31 | (-0.52, 0.73) | 1.01 | 997 | 442 |
|  | Baseline 25(OH)D† | 0.16 | 0.35 | (-0.51, 0.88) | 1 | 1295 | 417 |
|  | BMI† | -0.25 | 0.4 | (-1.03, 0.50) | 1 | 1124 | 435 |
|  | sigma | 0.43 | 0.52 | (0.05, 1.96) | 1.01 | 116 | 273 |
| Progressive sperm motility (n=5) (a) Weighted linear regression. (b) Bayesian meta-regression | | | | | | | Residual SE= 55.11 (df = 2),  Adjusted R² = 0.53,  F-statistic = 3.25 (df = 2,2), p = 0.235. |
| (a) | Variable | Coefficient (β) | SE | 95% CI | t-value | p-value |  |
|  | Intercept | 30.28 | 29.88 | (-91.82, 152.38) | 1.013 | 0.418 |  |
|  | Baseline 25OHD (ng/ml) | 1.21 | 0.51 | (-0.69, 3.12) | 2.376 | 0.141 |  |
|  | BMI (kg/m²) | -1.82 | 1.18 | (-5.82, 2.18) | -1.549 | 0.261 |  |
| (b) | Variable | Posterior Mean | Posterior SD | 95% CI | Rhat | Bulk ESS | Tail ESS |
|  | Intercept | 4.08 | 1.64 | (0.72, 7.44) | 1 | 1502 | 1065 |
|  | Baseline 25OHD (z) | 4.84 | 1.89 | (0.65, 8.25) | 1 | 1083 | 1183 |
|  | BMI (z) | -3.07 | 1.92 | (-6.72, 0.65) | 1 | 1024 | 1186 |
|  | sigma | 3.52 | 1.07 | (2.00, 6.29) | 1 | 912 | 1067 |


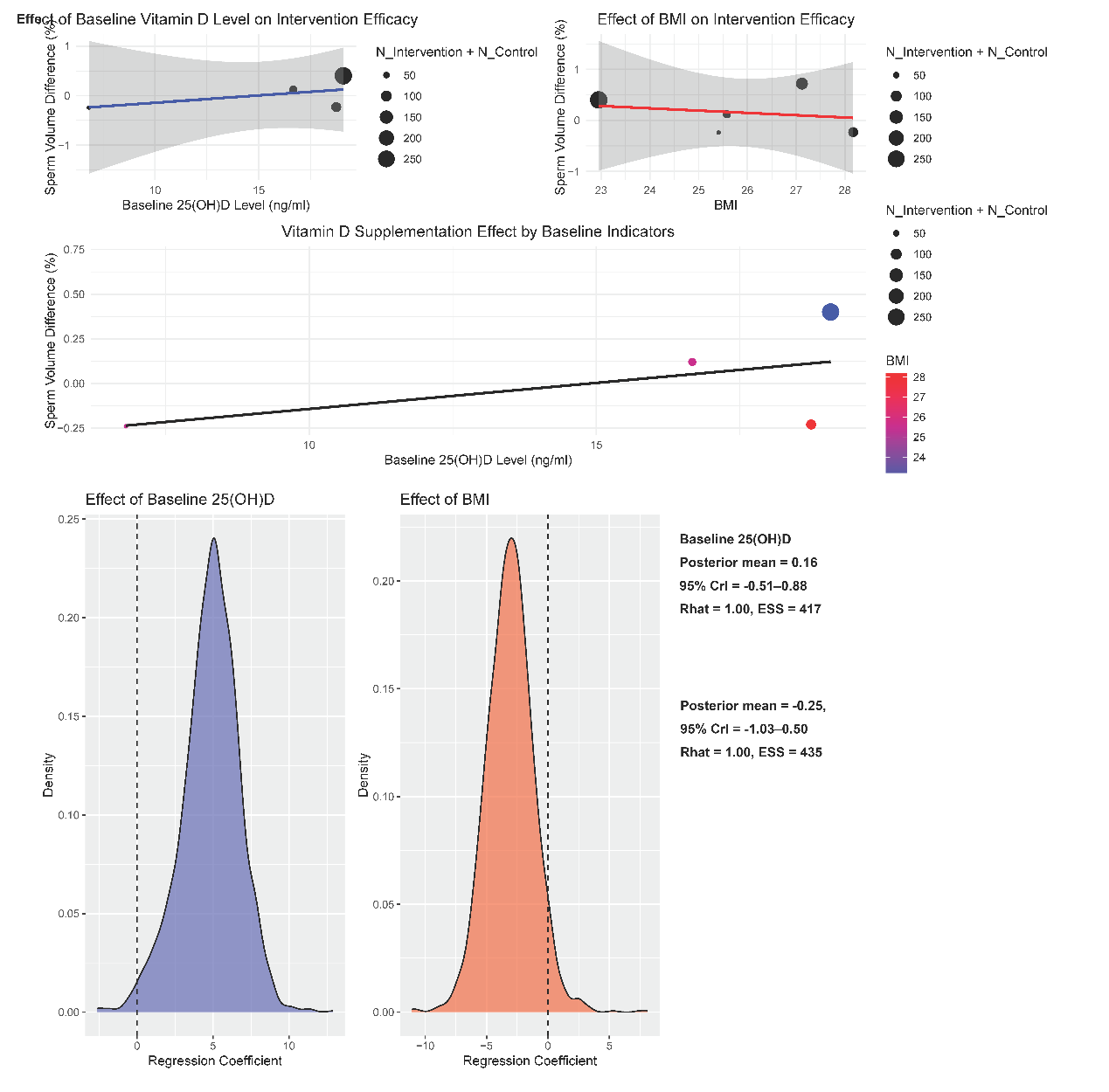


Figure S5. Meta-regression of semen volume based on baseline of 25(OH)D (ng/ml) and BMI (Kg/m^2^).

The traditional weighted linear regression analysis suggested that baseline 25(OH)D levels might be positively associated with semen volume, while BMI could be negatively associated.


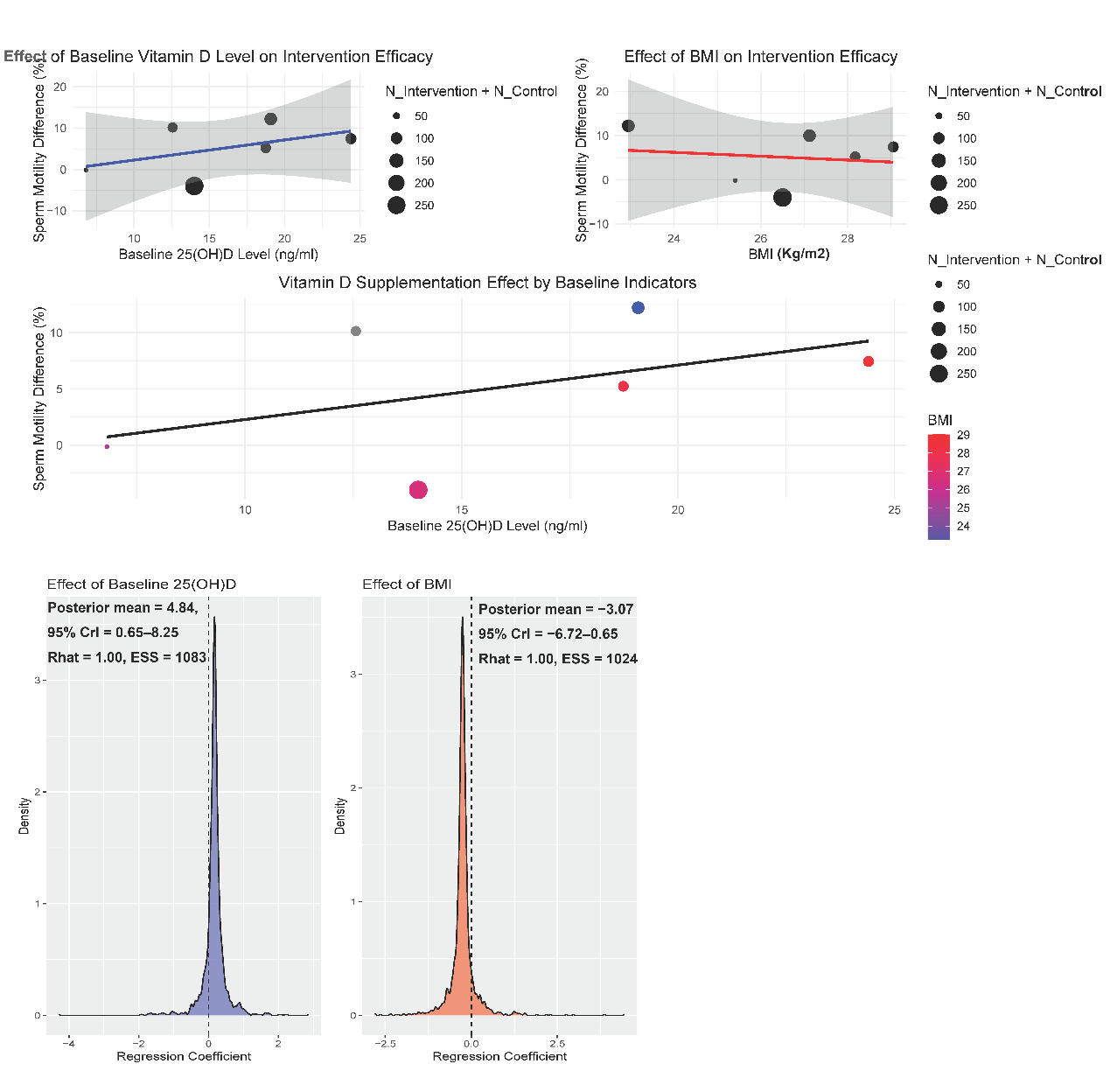


Figure S6. Meta-regression of progressive sperm motility based on baseline of 25(OH)D (ng/ml) and BMI (Kg/m^2^).

The Bayesian meta-regression analysis showed a significant positive association between baseline 25(OH)D levels and sperm motility, while the traditional method did not detect such an association.


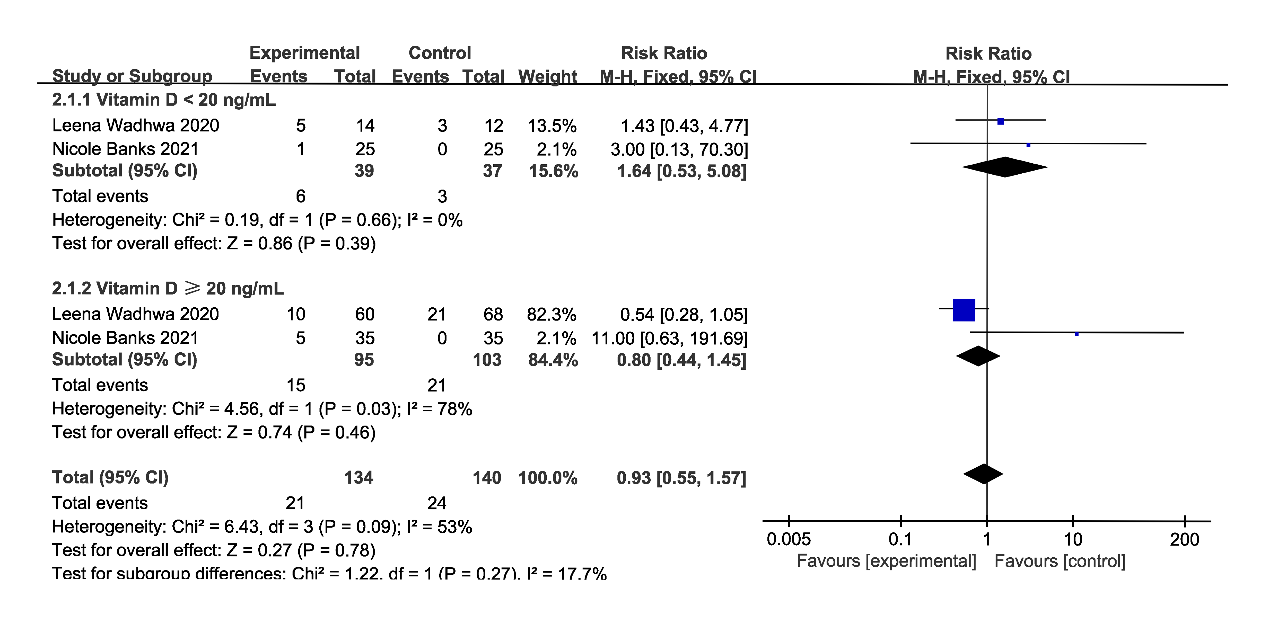


Figure S7. Subgroup analysis of clinical pregnancy rates by baseline vitamin D status.

Subgroup analyses stratified by participants' baseline VD levels demonstrated no statistically significant effect of VD supplementation on clinical pregnancy rates.


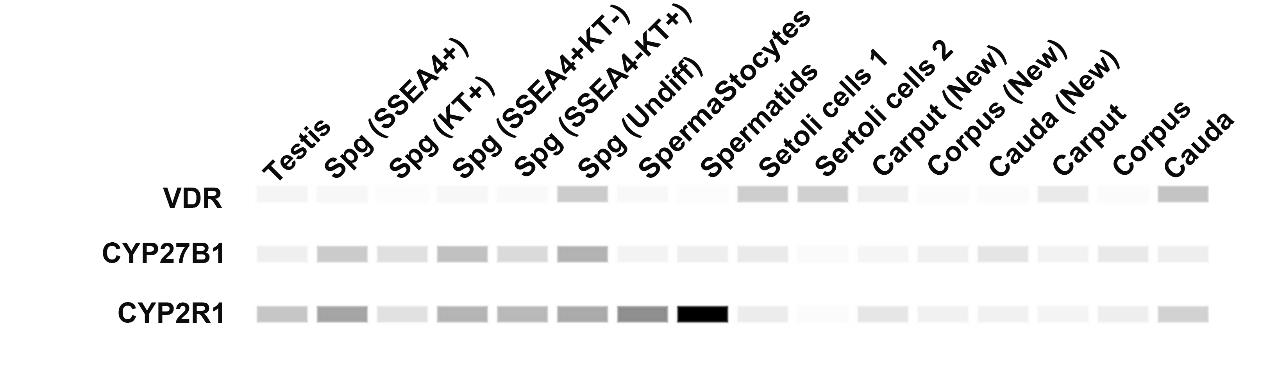


Figure S8. Expression of VDR, CYP27B1, and CYP2R1 in human testicular and epididymal tissues and cells. Notes: VDR (ENSMUSG00000111424), CYP27B1 (ENSMUSG00000111012), CYP2R1 (ENSMUSG00000186104).

RNA-seq data from the *MRGD V2* demonstrate that the **VDR,** CYP27B1, and CYP2R1A are expressed across multiple human testicular cell populations. Detectable VDR transcripts are observed in spermatogonia (SSEA4⁺, KIT⁺, and undifferentiated subtypes), spermatocytes, and Sertoli cells, indicating both germ-cell and somatic expression. In addition, expressions are also seen in the epididymis (caput, corpus, and cauda). These results provide molecular evidence that vitamin D signaling components are locally present in the male reproductive tract, supporting a potential role for VDR-mediated pathways in spermatogenesis and sperm maturation.
