# Supplementary figures and images for: Vitamin D supplementation in infertile men: a systematic review and meta-analysis of effects on semen quality and endocrine function
Source: PeerJ. 2026 Apr 13;14:e21002. doi: 10.7717/peerj.21002 (PMC13086023; doi:10.7717/peerj.21002)

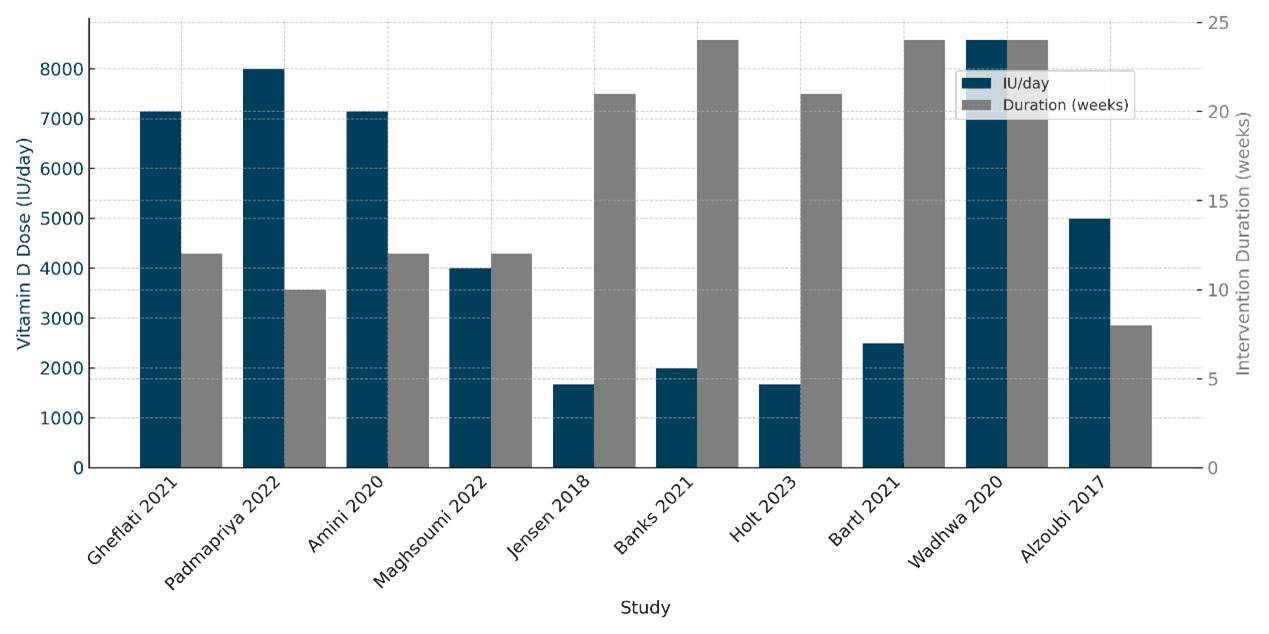

Supplement: Supplemental Information 5 [file peerj-14-21002-s005.png]

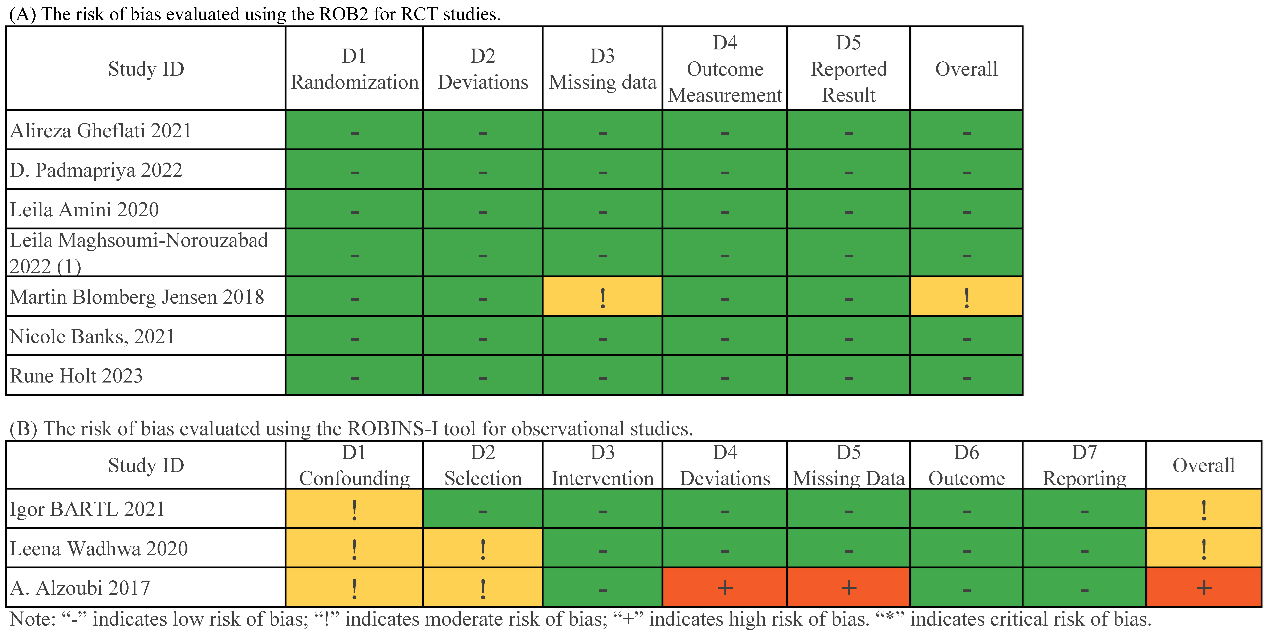

Supplement: Supplemental Information 6 — (A) ROB2 was applied for RCTs, and (B) ROBINS-I for observational studies. [file peerj-14-21002-s006.png]

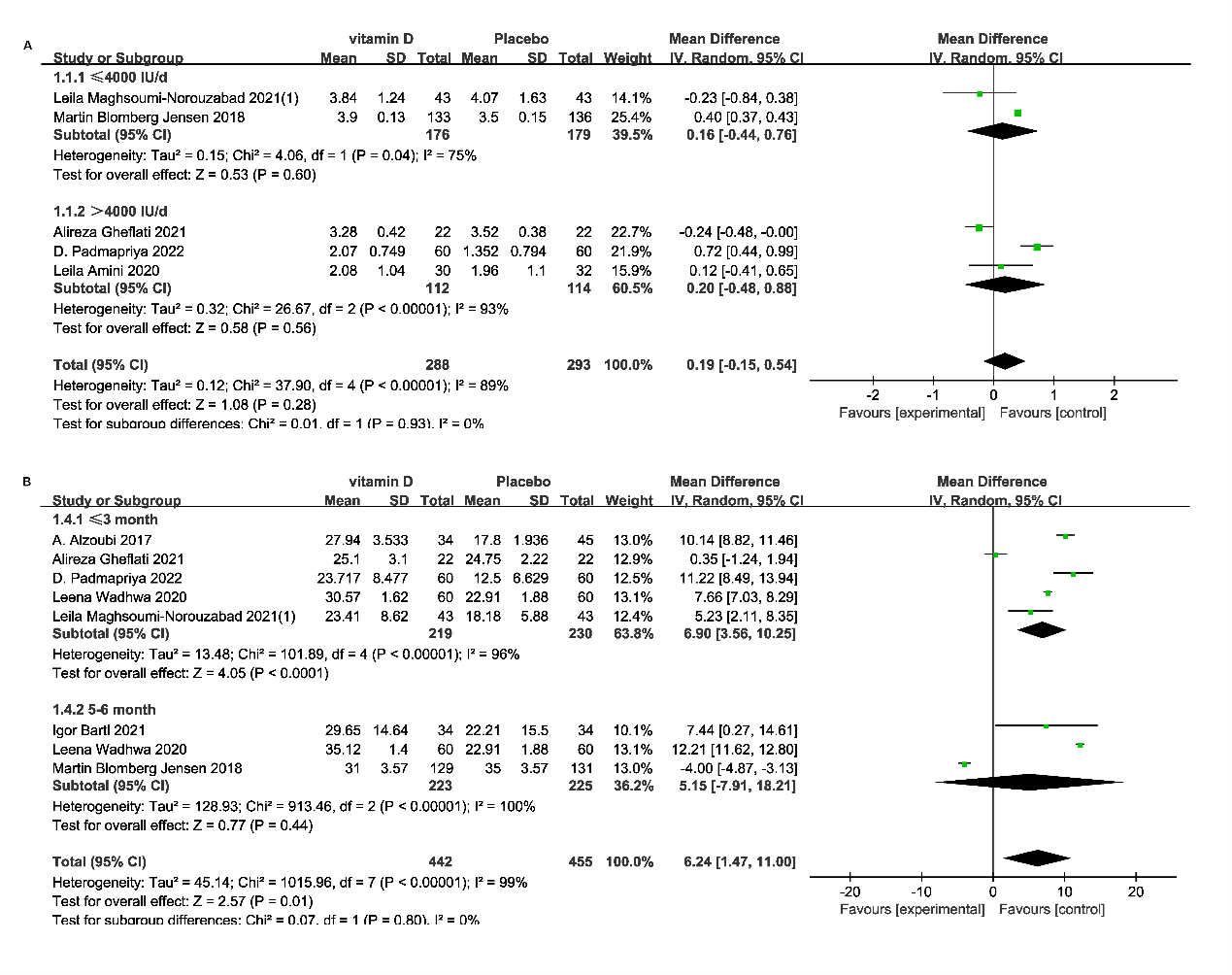

Supplement: Supplemental Information 7 — (A) Semen volume by intervention dosage. (B) Progressive sperm motility by intervention duration. [file peerj-14-21002-s007.png]

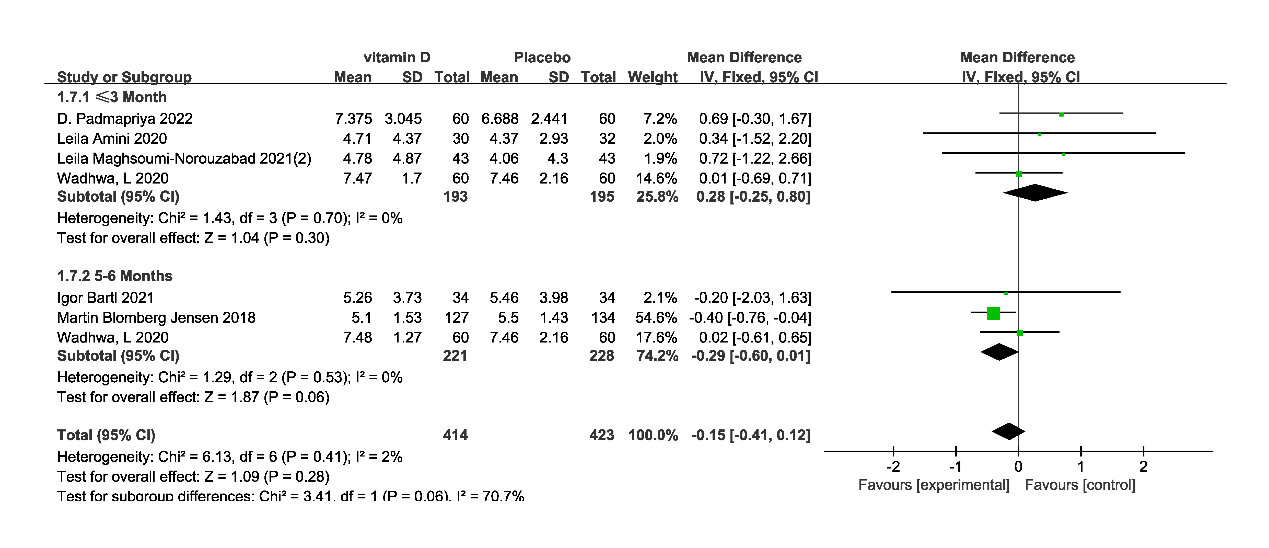

Supplement: Supplemental Information 8 — Subgroup analysis of FSH levels by treatment duration showed a borderline significant interaction (P = 0.06), with numerically divergent effects between longer (5–6 months, MD = –0.29 IU/L) and shorter (≤3 months, MD = 0.28 IU/L) interventions (P=0.06) . This finding suggests a potential benefit of longer-term vitamin D supplementation on FSH regulation. [file peerj-14-21002-s008.png]

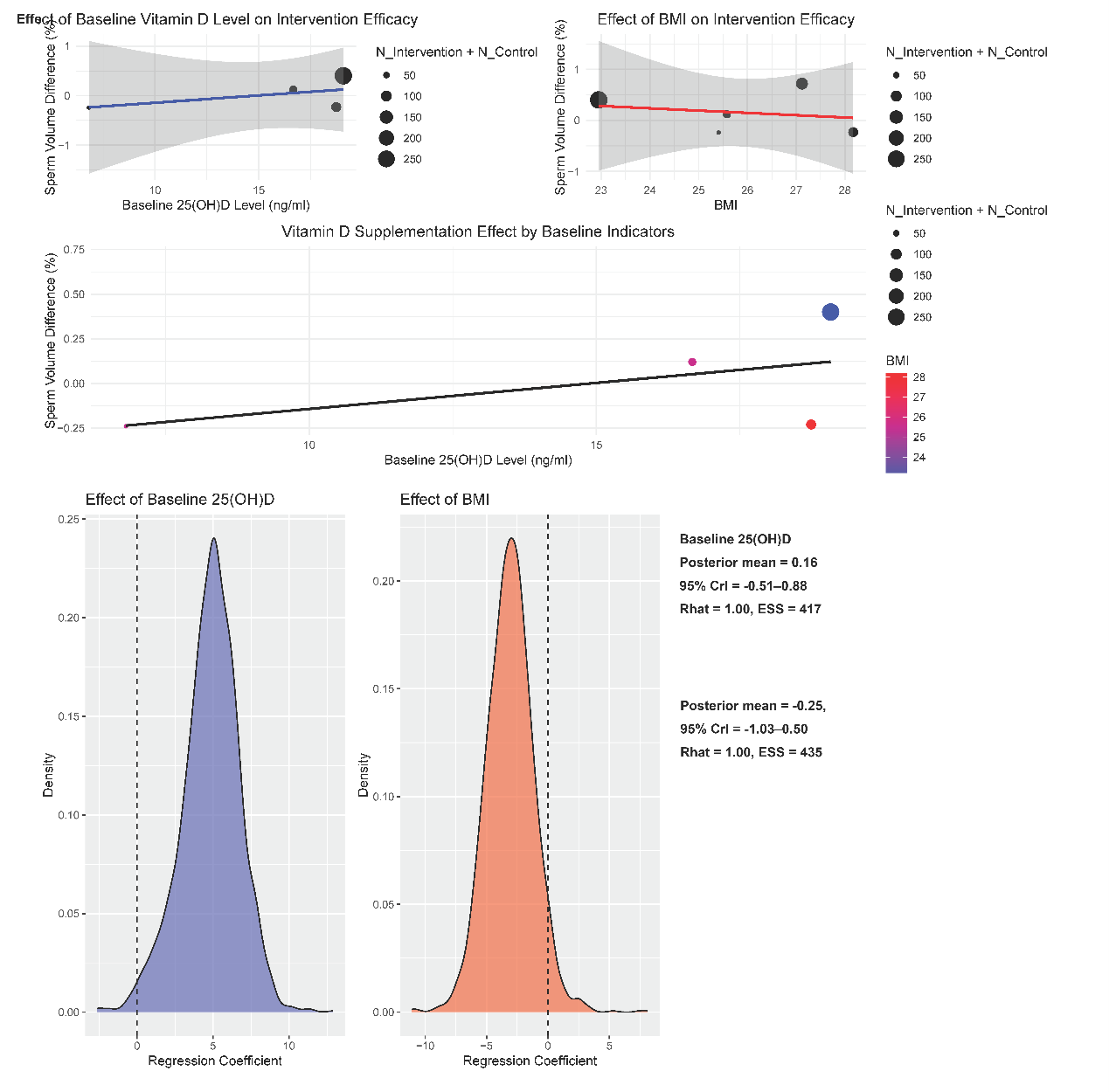

Supplement: Supplemental Information 9 — The traditional weighted linear regression analysis suggested that baseline 25(OH)D levels might be positively associated with semen volume, while BMI could be negatively associated . [file peerj-14-21002-s009.png]

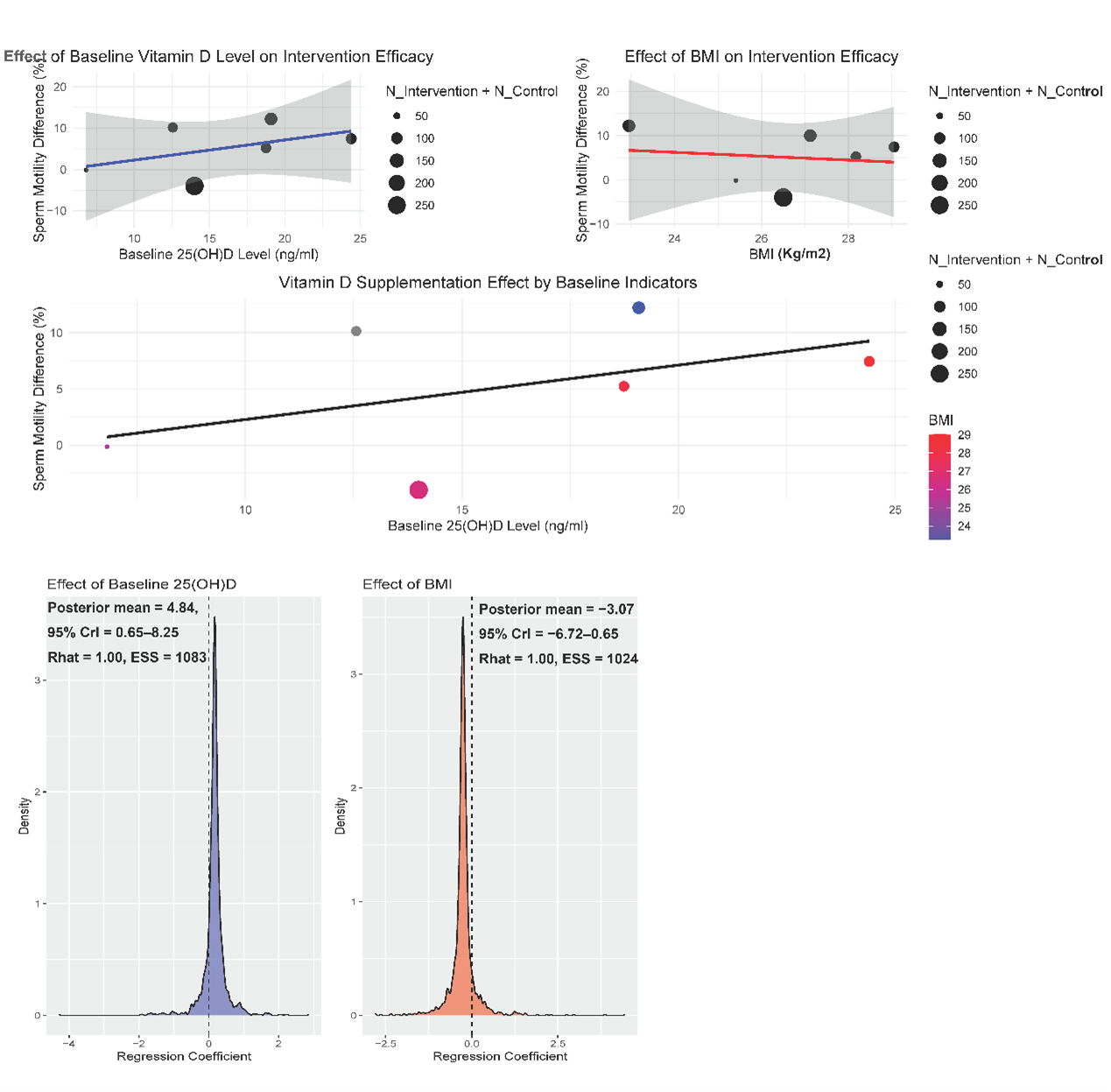

Supplement: Supplemental Information 10 — T he Bayesian meta-regression analysis showed a significant positive association between baseline 25(OH)D levels and sperm motility, while the traditional method did not detect such an association. [file peerj-14-21002-s010.png]

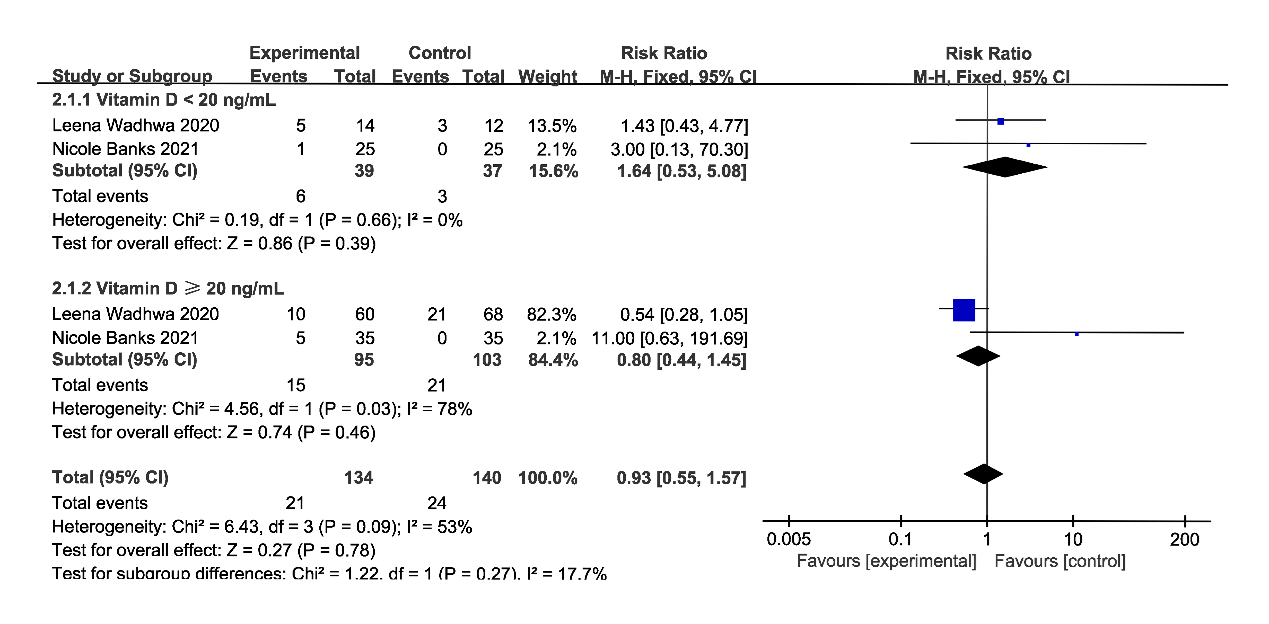

Supplement: Supplemental Information 11 — S ubgroup analyses stratified by participants’ baseline VD levels demonstrated no statistically significant effect of VD supplementation on clinical pregnancy rates . [file peerj-14-21002-s011.png]

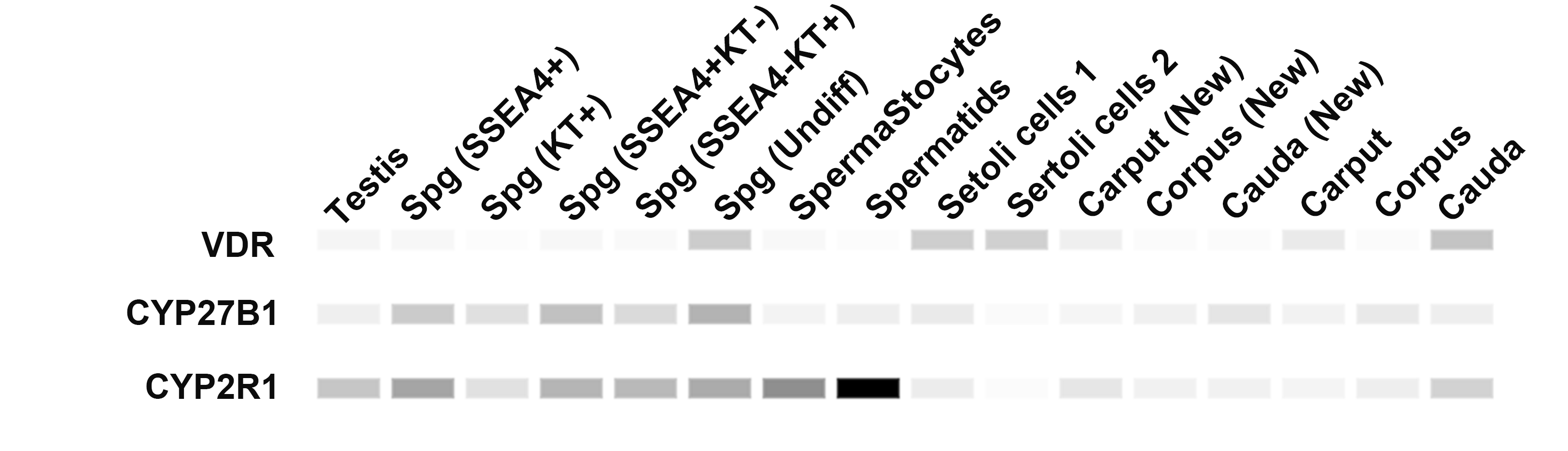

Supplement: Supplemental Information 12 — RNA-seq data from the MRGD V2 demonstrate that the VDR , CYP27B1, and CYP2R1A are expressed across multiple human testicular cell populations. Detectable VDR transcripts are observed in spermatogonia (SSEA4, KIT, and undifferentiated subtypes), spermatocytes, and Sertoli cells, indicating both germ-cell and somatic expression. In addition, expression s are also seen in the epididy mi s (caput, corpus, and cauda). These results provide molecular evidence that vitamin D signaling components are locally present in the male reproductive tract, supporting a potential role for VDR-mediated pathways in spermatogenesis and sperm maturation. [file peerj-14-21002-s012.png]

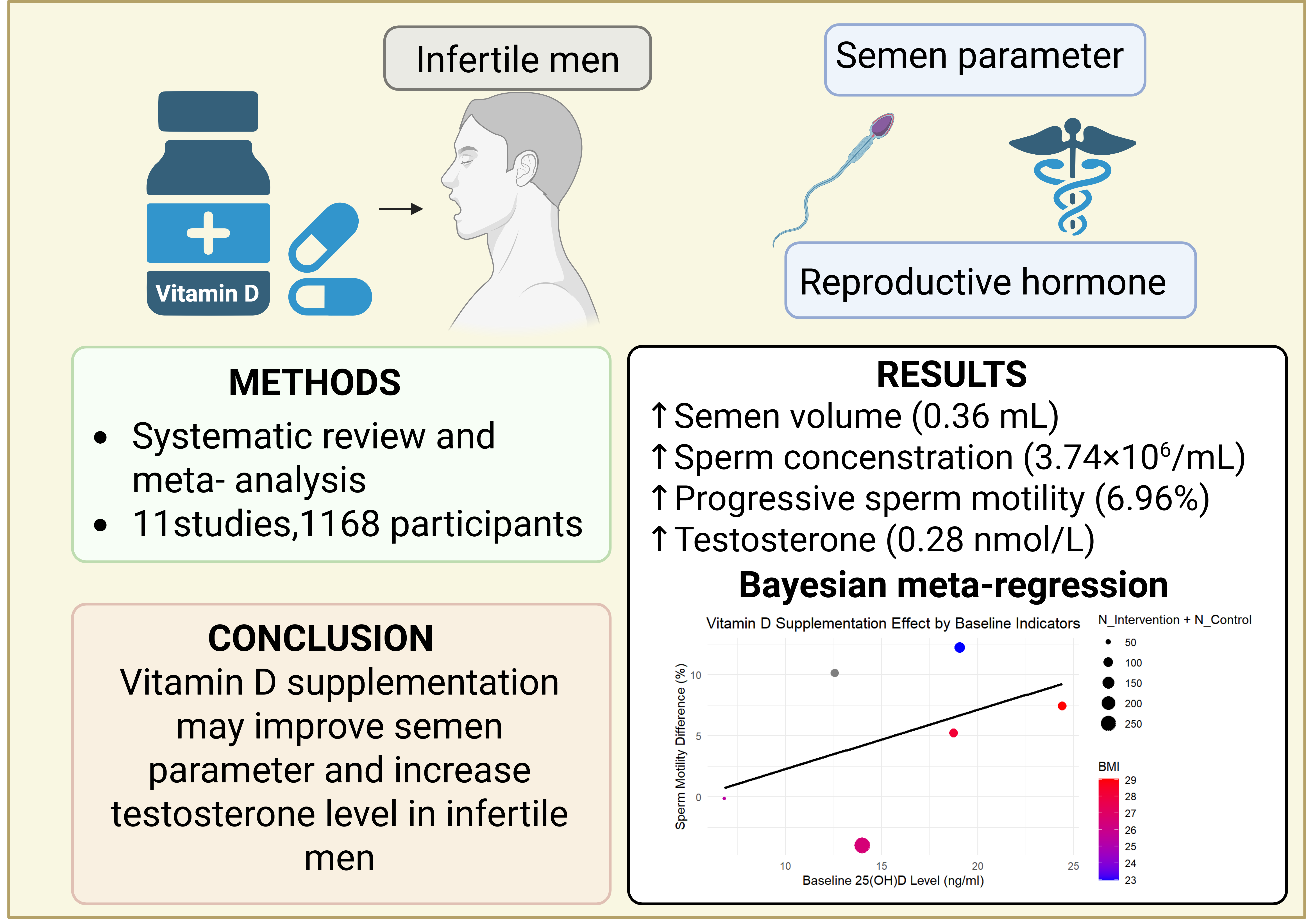

Supplement: Supplemental Information 13 — the findings indicated improvements in semen volume, sperm concentration, progressive sperm motility, and testosterone levels, suggesting a potential role of vitamin D supplementation in male infertility management. [file peerj-14-21002-s013.png]
